# Rapid turnover of DnaA at replication origin regions contributes to initiation control of DNA replication

**DOI:** 10.1371/journal.pgen.1006561

**Published:** 2017-02-06

**Authors:** Katrin Schenk, Ana B. Hervás, Thomas C. Rösch, Marc Eisemann, Bernhard A. Schmitt, Stephan Dahlke, Luise Kleine-Borgmann, Seán M. Murray, Peter L. Graumann

**Affiliations:** 1 SYNMIKRO, LOEWE Center for Synthetic Microbiology, Marburg, Germany; 2 Department of Chemistry, Philipps Universität Marburg, Marburg, Germany; 3 Department of Mathematics, Philipps Universität Marburg, Marburg, Germany; 4 Max Planck Institute for Terrestrial Microbiology, Marburg, Germany; A*STAR, SINGAPORE

## Abstract

DnaA is a conserved key regulator of replication initiation in bacteria, and is homologous to ORC proteins in archaea and in eukaryotic cells. The ATPase binds to several high affinity binding sites at the origin region and upon an unknown molecular trigger, spreads to several adjacent sites, inducing the formation of a helical super structure leading to initiation of replication. Using FRAP analysis of a functional YFP-DnaA allele in *Bacillus subtilis*, we show that DnaA is bound to *oriC* with a half-time of 2.5 seconds. DnaA shows similarly high turnover at the replication machinery, where DnaA is bound to DNA polymerase via YabA. The absence of YabA increases the half time binding of DnaA at *oriC*, showing that YabA plays a dual role in the regulation of DnaA, as a tether at the replication forks, and as a chaser at origin regions. Likewise, a deletion of *soj* (encoding a ParA protein) leads to an increase in residence time and to overinitiation, while a mutation in DnaA that leads to lowered initiation frequency, due to a reduced ATPase activity, shows a decreased residence time on binding sites. Finally, our single molecule tracking experiments show that DnaA rapidly moves between chromosomal binding sites, and does not arrest for more than few hundreds of milliseconds. In *Escherichia coli*, DnaA also shows low residence times in the range of 200 ms and oscillates between spatially opposite chromosome regions in a time frame of one to two seconds, independently of ongoing transcription. Thus, DnaA shows extremely rapid binding turnover on the chromosome including *oriC* regions in two bacterial species, which is influenced by Soj and YabA proteins in *B*. *subtilis*, and is crucial for balanced initiation control, likely preventing fatal premature multimerization and strand opening of DnaA at *oriC*.

## Author summary

Initiation of replication is a key event in the cell cycle of all living cells, and is mediated by the ATPase DnaA in bacteria, and by ORC proteins in eukaryotic cells. DnaA binds to several high affinity binding sites at the origin region of replication (*oriC*) on the bacterial chromosome, triggers the unwinding of the DNA duplex nearby, and additionally supports loading of the DNA helicase, which in turn leads to the establishment of the DNA replication machinery. How the binding of DnaA to *oriC* and the triggering of duplex opening are regulated is under extensive investigation. Using two different fluorescence microscopy techniques, we show that DnaA binding and unbinding to *oriC* is very rapid in two bacterial species and occurs in the range of few seconds. Moreover, DnaA binds to several additional sites on the chromosome, but with an even shorter binding half-time than at *oriC*: average residence time throughout the chromosome is about 200 ms, as determined by single molecule microscopy. In the absence of two negative regulators, YabA and Soj, DnaA in *Bacillus subtilis* binds longer to *oriC* and to other sites on the chromosome, accompanied by a higher frequency of initiation per cell cycle, whereas the expression of a DnaA mutant protein that shows even faster exchange rates results in decreased initiation frequency. Our data reveal that DnaA exchanges rapidly at *oriC*, and that tight regulation of turnover is important for proper initiation control. We also show that YabA has a dual role, a) in tethering DnaA to the replication machinery and restricting its mobility within the cell and b) in increasing DnaA turnover at *oriC*, both of which activities reduce the risk of reinitiation during later stages in the cell cycle.

## Introduction

All cells must be able to integrate environmental and internal physiological cues into the decision when to commence the duplication of the genome in order to initiate the proliferation cycle. Nature appears to have invented the process of replication initiation only once, because the key players, called ORC in eukaryotic and in archaeal cells, and DnaA in bacteria, are conserved AAA (ATPases Associated with diverse cellular Activities) proteins, whose ATPase activity leads to conformational changes that are transduced into mechanical force or into switch-like processes. For DnaA, ATP hydrolysis confers a crucial role in initiation control [1, 2]. DnaA has been shown to associate with several high affinity binding sites at the origin region on the circular chromosome and upon an unknown molecular trigger, to extend binding to several adjacent low-affinity binding sites, which induces the formation of a helical, right handed polymeric structure [2–4]. Superhelical torsional stress of this superstructure leads to strand opening at an adjacent AT rich region, which is stabilized by an indispensable repeating trinucleotide motif term the “DnaA-trio”, to which DnaA binds with its ssDNA binding region [37]. Subsequently, DnaA interacts with helicase loader proteins to achieve the establishment of replication forks via loading of helicase and further replication proteins [5, 6]. The time point of initiation of replication starts the cell cycle, and must be in tune with extracellular stimuli as well as with the energetic state of the cell. A failure to limit initiation of replication to once per cell cycle leads to growth defects and in severe cases ultimately to lethality.

The activity and regulation of DnaA has been described in most detail in *Escherichia coli*, where several negative regulatory systems cooperate to limit the generation of an initiator complex to once per cycle [5, 6]. Key aspects are the sequestration of *oriC* regions based on hemimethylation, the activation of ATPase activity of DnaA via active replication forks and sequestration of free DnaA to a sink of DnaA-binding sites that are duplicated soon after replication initiation. Several of the regulatory proteins described in *E*. *coli* are restricted to enteric bacteria, and are not found in most other species. In *Bacillus subtilis*, a model organism for Gram positive bacteria, two negative regulators of DnaA activity are known, YabA and Soj (a ParA type protein) [7]. YabA is a tetrameric protein that serves as an adaptor to recruit DnaA to DnaN, the sliding clamp of DNA polymerase [8]. It has been speculated that DnaA is thereby sequestered from origin regions after initiation of replication, consistent with the finding that DnaA is more dispersed in *yabA* deleted cells, and is visible at *oriC* regions in mutant cells to a larger extent than in wild type cells [9]. YabA has also been shown to a) be associated with *oriC* DNA *in vivo*, and b) to interfere with cooperative DNA binding of DnaA *in vitro* [10, 11]. During the cell cycle, YabA is mostly present at the replication forks [8, 12]. Therefore, it is not clear how and where YabA exerts its function in restricting the activity of DnaA *in vivo*. Soj protein is another regulator of DnaA, which binds directly to the initiator protein [13]. Dependent on whether Soj has bound ATP, and is in a dimeric state, or ADP, in a monomeric state, it either activates DnaA or inhibits its activity by interfering with the multimerization of DnaA [14], a key step in initiation of replication at *oriC* [15]. Overall, Soj acts as a negative regulator, because a *soj* deletion leads to increased initiation events during the cell cycle [16].

We wished to understand the interaction of DnaA with *oriC* and the replication machinery *in vivo* in more detail, and to investigate the connection between DnaA and its two regulators. We therefore employed fluorescence recovery after photobleaching (FRAP) and single molecule fluorescence microscopy to find that DnaA association at *oriC* is short-lived, and upon only moderate elongation of its dwell time, i.e. in the absence of one of its negative regulators, overinitiation takes place, revealing that initiation control occurs every few seconds in bacteria, and is thus extremely dynamic.

## Results

### YabA and DnaA co-localize with origin regions and replication forks throughout the cell cycle

**Fig 1:**
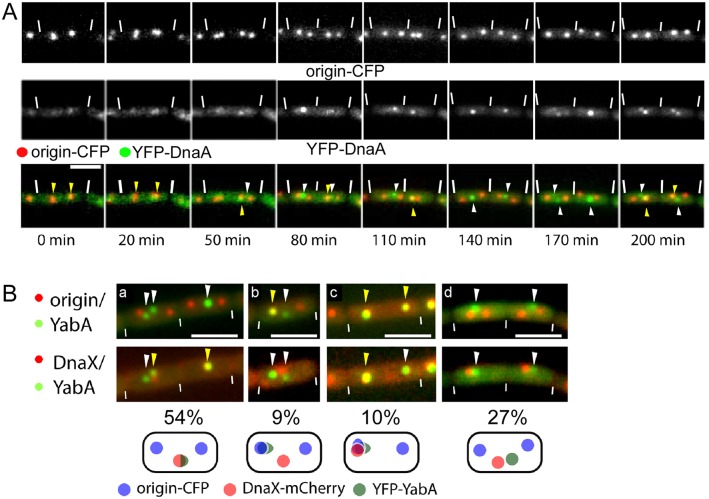
A) Time lapse of YFP-DnaA (strain ME15) localization with respect to the position of the origin of replication. Upper panel: CFP channel showing the position of origin regions marked by LacI-CFP (note that in the first frame, the signals most likely correspond to non-segregated origin regions), middle panel: localization of YFP-DnaA, lower panel: overlay of YFP-DnaA signals (green) with the origin of replication (red) over a time course of 200 min. Triangles indicate the position of YFP-DnaA signals, with yellow triangles indicating co-localization and white triangles no co-localization of both signals. White lines, cell borders; scale bar, 2 μm. Cells were grown in S750 minimal medium at 30°C until early exponential phase. Then the expression of YFP-DnaA was induced with 0.1% xylose (w/v) 15 min. B) Fluorescence microscopy showing YFP-YabA localization patterns in exponentially growing *B*. *subtilis* cells. Overlays of YFP-YabA signals (green) with origin-CFP (red) in the upper panel and with DnaX-mCherry (red) in the lower panel. Triangles indicate positions of YFP-YabA foci: white triangles no co-localization with origin or replication machinery, yellow triangles co-localization. White lines, cell borders; scale bars, 2 μm. Numbers represent percentage of observed pattern in a sample of 208 cells (note that panel d also includes 10% of cells in which one or two of the three signals was not visible). Cartoons below image panels illustrate the four different localization pattern described.

DnaA has been shown to be present at the origin of replication region(s) early in the cell cycle, but predominantly at the replication machinery during most of the cell cycle, after duplicated origin regions have been separated towards opposite cell poles [9]. DnaA can be expressed as a fully functional N-terminal YFP fusion protein in *B*. *subtilis* [9], and as a sandwich fluorescent protein fusion in *E*. *coli* [17, 18], as the sole source of DnaA in the cell. The expression level of YFP-DnaA driven by the xylose promoter was adjusted to be similar than that of DnaA driven by its original promoter in *B*. *subtilis* (S1A Fig), allowing us to follow its localization in live cells throughout the cell cycle. Previously, we have performed statistical localization studies of YFP-DnaA, showing that DnaA mostly co-localizes with the clamp loader complex of the replication machinery (visualized through DnaX-CFP), after the origin regions have separated towards opposite cell poles [9]. Fig 1A shows an example of a time lapse experiment of YFP-DnaA (original locus, CDS7, Table 1) in a large cell that has initiated replication before the next cell cycle (this is frequently the case under the growth conditions used in this study, t_D_ = 92 min, [9]), where DnaA co-localizes with *oriC* regions for 20 minutes. When duplicated origins have separated, a single YFP-DnaA focus remains between origins in the left cell half, likely co-localizing with the central replication machinery, while in the right cell half, DnaA remains at one origin region for some time. From minute 140 to minute 170, DnaA is no longer seen at origin regions, while at minute 200, two origins show YFP-DnaA foci, with a central DnaA focus remaining. This example shows that DnaA can be seen to accumulate at replication forks as well as at origin regions after replication has been initiated (as judged from the separation of duplicated origin regions). We were only able to obtain ten complete time lapse experiments with YFP-DnaA foci being visible at all time intervals, not permitting us to make any statistical statements, but the data show that DnaA has the capacity to visibly move between replication machinery, cytosol and origins during the cell cycle. Thus, although a fraction of DnaA is tethered to the replication machinery throughout most of the cell cycle, DnaA also accumulates at *oriC* after replication initiation, which could potentially cause unwanted reinitiation events. Therefore, additional control mechanisms must exist that prevent reinitiation at *oriC*, which include the two negative DnaA regulators, Soj (ParA) and YabA.

**Table 1:** Strains.

Strain	Genotype (resistance)	Reference
*E*. *coli*		
MG1655	*dnaA-eyfp*	[17]
TCR361	pHGWA P_T7_::*his*6-*mNeonGreen* (*amp*^R^)	this study
*B*. *subtilis*		
PY79	*wild type*	
PG208	*spo0J(359°)*::*lacO cassette* (cm^R^) *thrC*::*lacI-cfp* (mls^R^) (“*origin-cfp”*)	[53]
JDS190	*amyE*::*P*_*xyl*_*yfp-yabA* (spec^R^)	this study
PG1037	*P*_*xyl*_ *yfp-dnaA* (cm^R^)	[9]
AG1505	*Δsoj-spo0J*::*spec*	[54]
CDS7	*P*_*xyl*_ *yfp-dnaA* (tet^R^), origin-CFP (cm^R^, mls^R^)	
AHV2	*amyE*::*P*_*xyl*_*yfp-dnaA* (spec^R^)	this study
ME15	*origin-cfp* (cm^R^, mls^R^) *amyE*::*yfp-dnaA* (spec^R^)	this study
ME20	*origin-cfp* (cm^R^, mls^R^) *amyE*::*yfp-dnaAE183Q* (spec^R^)	this study
ME21	*origin-cfp* (cm^R^, mls^R^) *amyE*::*yfp-dnaAR387C* (spec^R^)	this study
KS114	*dnaX-mcherry* (tet^R^)	this study
KS119	*origin-cfp* (cm^R^, mls^R^) *amyE*::*yfp-yabA* (spec^R^)	this study
KS140	*origin-cfp* (cm^R^, mls^R^) *amyE*::*yfp-yabA* (spec^R^) *dnaX-mcherry* (tet^R^)	this study
KS167	*ΔyabA*::*phleo amyE*::*yfp-yabA* (spec^R^, xyl)	this study
KS171	*ΔyabA*::*phleo P*_*xyl*_ *yfp-dnaA* (cm^R^)	this study
KS173	*ΔyabA*::*phleo amyE*::*yfp-yabA* (spec^R^, IPTG)	this study
KS175	*Δsoj-spo0J*::*spec P*_*xyl*_ *yfp-dnaA* (cm^R^)	this study
KS192	*Δsoj-spo0J*::*spec amyE*::*yfp-dnaA* (kan^R^, xyl)	this study
NEJ100	*amyE*::*spo0J-yfp* (spec^R^, xyl)	this study

YabA has been reported to largely co-localize with the replication machinery [8, 12]. On the other hand, *in vivo* and *in vitro* data show that YabA can influence binding of DnaA to *oriC* sequences [5, 6, 10], throughout the cell cycle, and to affect multimerization of DnaA [19]. We wished to address the question how YabA can affect *oriC* binding of DnaA when based on epifluorescence microscopy YabA is visually absent from *oriC* during most of the cell cycle [8, 12]. We therefore visualized a functional [8, 12] YFP-YabA fusion throughout the cell cycle, examining its co-localization with both, *oriC* regions, and with DnaX as a marker for the replication machinery (*yfp-yabA*, *lacI-cfp*/*lacO* array at *oriC*, *dnaX-mCherry*). Fig 1B shows that in 54% of cells, YabA co-localizes with the central DnaX focus, and is not visible at bipolar *oriC* regions. In 10% of cells, all three markers co-localize (initiation) and in 9% of cells, YabA co-localizes with DnaX and with one of the two *oriC* regions. In the remaining cells (27%), a YabA focus is present, but does not co-localize with either *oriC* or DnaX. Therefore, YabA can also visibly accumulate at *oriC* during ongoing replication. We analysed the localization pattern of YFP-YabA more precisely, grouping cells according to cell length, which correlates with the age of the cell. S2 Fig shows that in small, medium sized and large cells, YabA is present at the replication forks and not at *oriC* regions in about 50% of cells (panels B, D and G), and co-localizes with *oriC* in 10% of young cells, in 6% of medium-age cells, and in 13% of old cells (panels C, F and H). Interestingly, YabA does not co-localize with either *oriC* or DnaX in about 25% of cells, possibly interacting with DnaA that is bound at promoter regions on the chromosome. YabA was seen to co-localize with only one of two origins in 9% of middle aged cells (S2 Fig, panel E), showing that *oriC* localization can be asymmetric. Therefore, YabA largely follows the localization pattern of DnaA [9] (Fig 1A), and a considerable fraction of YabA can be asymmetrically positioned, both at the forks and at *oriC*. The patterns of localization of DnaA and of YabA could be explained if they were to alternate between binding at *oriC* and at replication forks in a frame of few minutes or even less, which is shown below.

### DnaA shows rapid turnover at *oriC* and at the replication machinery

**Fig 2:**
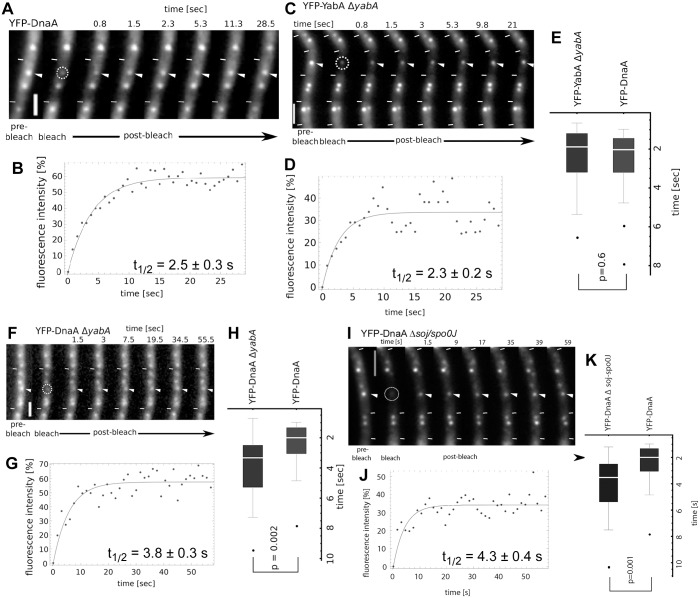
FRAP analysis of exponentially growing cells. A) FRAP sequence of cells expressing YFP-DnaA as sole source of the protein (strain PG1037). Recovery of the fluorescence signal in the region of interest (dashed circle) over time is indicated by the white triangle. White lines, cell borders, scale bar 2 μm. B) Fluorescence intensity (%) plotted over time (s). The line represents the fit used to calculate recovery half-time. Diagram displays data obtained from a single experiment shown in (A). Calculated recovery half-time for YFP-DnaA determined from 32 experiments is 2.5 ± 0.3 s. C) FRAP sequence of YFP-YabA expressed in a *yabA* deletion strain (KS173), similar to panel A). D) Fluorescence intensity (%) plotted over time (s), similar to panel B), determined from 41 experiments. E) Box-plot showing the distribution of calculated recovery half-times for YFP-DnaA (PG1037) and YFP-YabA (KS173). Black dots are considered outliers (lie outside of 1.5 ITR). YFP-YabA and YFP-DnaA displayed a similar dynamic behavior (students t-test, p = 0.6). F) FRAP sequence of cells expressing YFP-DnaA in the absence of YabA (strain KS171), similar to panel A. G) Fluorescence intensity (%) plotted over time (s), for strain KS171 (n = 32), same as panel B. H) Box-plot showing the distribution of calculated recovery half-times for YFP-DnaA (PG1037) and of YFP-DnaA in cells lacking YabA (KS171). I) FRAP sequence of cells expressing YFP-DnaA in the absence of Spo0J/Soj (strain KS175), similar to panel A. J) Fluorescence intensity (%) plotted over time (s), for strain KS175 (n = 42), same as panel B. K) Box-plot showing the distribution of calculated recovery half-times for YFP-DnaA (PG1037) and of YFP-DnaA in cells lacking Spo0J/Soj (KS175).

To shed light on the connection between YabA, DnaA and binding to origin regions, we determined the turnover rate of YFP-DnaA binding to *oriC*, using FRAP analysis. Large cells containing two YFP-DnaA foci usually contain YFP-DnaA co-localizing with *oriC* regions, because replication has terminated [9], and were therefore used for the analyses. From 32 experiments, we found a half time recovery for YFP-DnaA of 2.5 ± 0.3 seconds (YFP-DnaA original locus, strain PG1037, Table 2); for a typical FRAP experiment see Fig 2A. Therefore, turnover of DnaA binding is extremely rapid. In contrast, LacI binds to the *lacO* site for about 4 minutes on average [20], and TetR has a half-time recovery of 2 to 2.5 minutes on a *tetO* array in eukaryotes [21, 22]. When we repeated experiments using LacI-GFP binding to a *lacO* array (the “origin tag”) in *B*. *subtilis*, we determined a half-time recovery of 2.4 ± 0.58 minutes for LacI-GFP binding (S3A and S3B Fig), which is quite similar to the recovery of the *tetO* array/TetR-GFP system. By normalizing the FRAP data by the whole cell intensity (see Material and methods), we determined if a fraction of DnaA molecules remain bound to *oriC* on a timescale much longer than that of the experiment (i.e. if a fraction of DnaA does not exchange during the course of the experiment). From Table 2, it can be deduced that the stationary fraction of YFP-DnaA at *oriC* is about 10% (strain PG1037) and is therefore very low, indicating that most DnaA molecules constantly turn over at this chromosomal site. In some cells, there was no measurable stationary fraction, while in others it was about 20%, indicating considerable heterogeneity in this aspect during the cell cycle, although it must be kept in mind that FRAP analyses are inherently noisy. To rule out that FRAP recovery is affected by even a possible slight overproduction of DnaA, we grew cells in the presence of 0.1% xylose, leading to reduced levels of YFP-DnaA (S1A Fig). Half-time recovery in these cells was 2.39 ± 0.85 s (S3C Fig), showing that our experiments are not affected by unphysiological levels of YFP-DnaA.

**Table 2:** FRAP analysis.

Strain	Recovery half time (s)	SEM	n	Immobile fraction (%)	SEM
PG1037 (yfp-dnaA*)	2.5	0.3	32	10.4	2.0
AHV2 (*amyE*::*P*_*xyl*_*yfp-dnaA*)	2.9	0.2	61	n.d.	n.d.
ME15 (*amyE*::*yfp-dnaA*, *oriC-CFP*)	2.7	0.5	61	10.3	1.2
KS175 (yfp-dnaA*, Δ*soj-spo0J*)	4.3	0.4	42	14.6	1.4
KS171 (yfp-dnaA*, Δ*yabA*)	3.8	0.3	32	10.8	1.2
ME21 (*amyE*::*yfp-dnaAR387C*)	2.5	0.4	33	9.1	2.1
JDS190 (*amyE*::*yfp-yabA*)	2.3	0.2	41	n.d.	n.d.
KS173 (*amyE*::*yfp-yabA*, Δ*yabA*)	2.3	0.2	36	19.7	2.1

*expressed as sole source of the protein from original gene locus

We wished to determine if ectopically expressed YFP-DnaA behaves in a similar manner as the fusion expressed from the original gene locus. Ectopically induced YFP-DnaA reduces the expression of wild type DnaA through auto-repression of DnaA at the *dnaA* promoter [23], which we verified by Western blotting (S1B Fig). We expressed YFP-DnaA for 30 min with 0.1% xylose (w/v) prior to FRAP experiments. Under this condition, somewhat more YFP-DnaA than non-tagged DnaA was present within the cells, but not exceeding the amount of DnaA in cells lacking ectopically expressed YFP-DnaA (S1B Fig, lane 4). Importantly, half time FRAP recovery was similar for ectopically expressed YFP-DnaA (2.9 ± 0.2 s, n = 61) (strain AHV2, Tables 1 and 2), compared with YFP-DnaA expressed from the original gene locus, showing that YFP-DnaA turns over similarly in the presence of wild type DnaA in a merodiploid strain, compared to being the sole source of DnaA in the cell.

To ensure that FRAP recovery reflects YFP-DnaA bound to *oriC*, we analysed recovery in strain ME15, which ectopically expresses YFP-DnaA and carries an origin-CFP tag (Table 1). From 10 experiments, we found a recovery time for YFP-DnaA co-localizing with *oriC* of 2.7 s ± 0.5 s (S4A and S4C Fig, Table 2), showing that the FRAP data adequately describe the turnover of YFP-DnaA at *oriC*.

**Fig 3:**
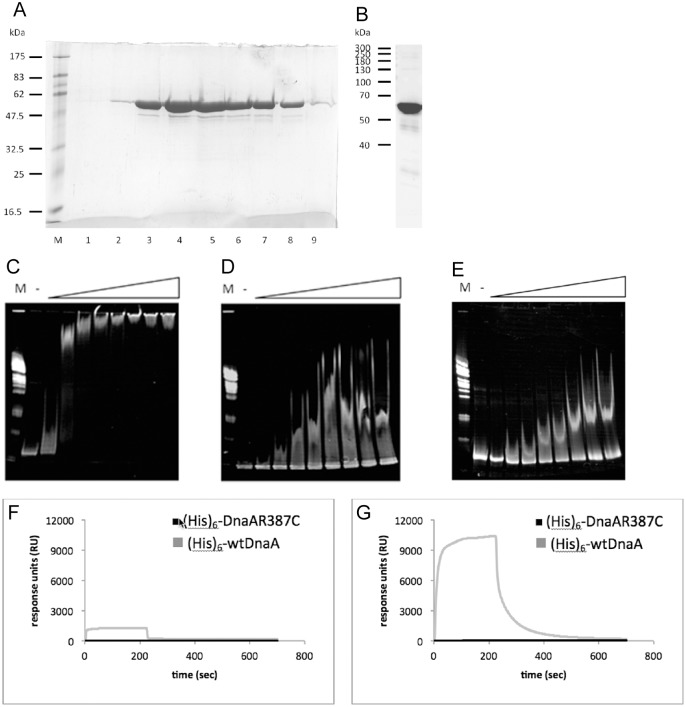
Biochemistry of DnaA. A) Coomassie-stained SDS PAGE showing the purification of DnaA by gel filtration chromatography following nickel NTA affinity chromatography; shown are peak elution fractions. B) Western blot analysis using anti DnaA antiserum of purified DnaA, showing that lower bands in gel from panel A) are DnaA degradation products. C) EMSA (ethidium-bromide stained 4–12% gradient acrylamide gels) of wild type DnaA binding to a 624 bp fragment containing *oriC* (0.9 pmol). M = marker,— = no protein, following lanes increasing amounts of DnaA, of 2, 4, 8, 16, 20, 40, 60 and 80 pmol. D) EMSA of wild type DnaA binding to a 532 bp fragment not containing *dnaA* boxes, same as panel C). E) EMSA of DnaAR387C binding to a 624 bp *oriC* DNA fragment, same as panel D). F) Surface plasmon resonance [SPR] experiments with DnaAR387C [2.5 μM] binding to double-biotinylated [5′ and 3′ ends] 624 bp *oriC*-DNA [0.25 pmol] that is bound to a streptavidin coated sensor chip surface. The protein sample [75 μl] was loaded to the chip at a flow rate of 20 μl/min, i.e. for 225 seconds, followed by protein dissociation from the DNA. Protein-DNA interactions were measured as response units [RU] in real time over a period of 700 seconds. All values represent the difference of absolute measured response units that have been subtracted from unspecific binding events in a control flow chamber. Black line: DnaAR387C, grey line: wtDnaA. G) SPR in the presence of 2.5 mM ATP.

**Fig 4:**
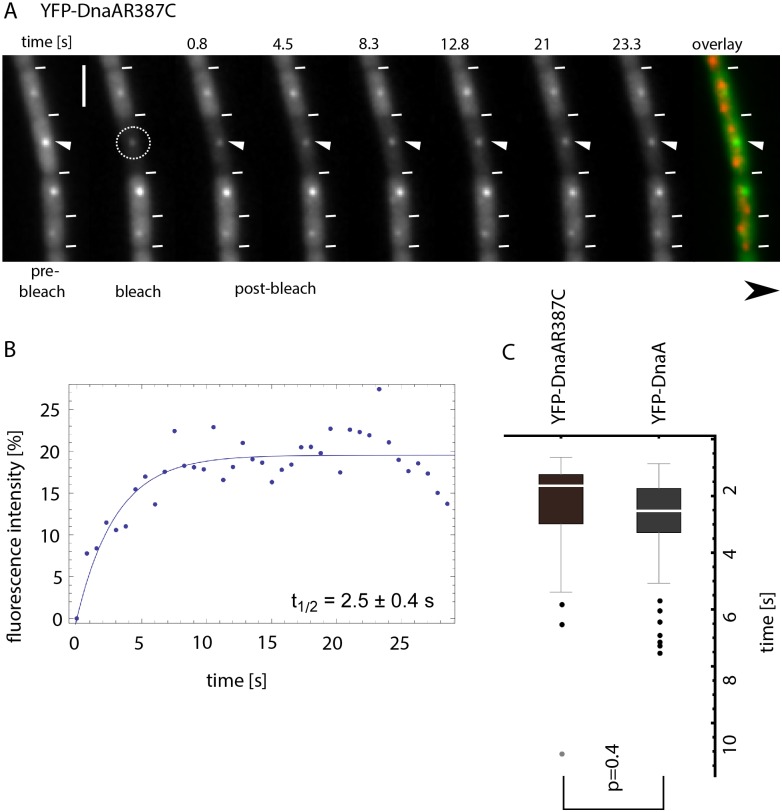
FRAP measurements of YFP-DnaAR387C. A) FRAP sequence showing the recovery of YFP-DnaAR387C fluorescence signal after bleaching. White triangle, area of interest; white dashed circle, bleached area. White lines, cell borders; scale bar, 2 μm. B) Fluorescence intensity plotted over time. Blue line, fit. Recovery half-time is 2.5 ± 0.4 s (n = 33). C) Box-plot showing calculated recovery half-times for YFP-DnaA (ME15) and YFP-DnaAR387C (ME21). Black dots are considered outliers and grey dots extreme values (lie outside of 1.5 ITR or 3 ITR, respectively). No significant difference between both half-times could be detected (p = 0.4, students t-test).

To find out if turnover of DnaA at *oriC* differs from that at the replication machinery, we performed FRAP analysis of YFP-DnaA foci not co-localizing with *oriC* (S4B and S4D Fig). This fraction of foci largely represents replication fork-bound DnaA, and has a half-time turnover that was very similar to that of *oriC*-localized YFP-DnaA (3.08 ± 0.5 s, n = 10). To further substantiate this point, and to investigate the localization pattern of a DnaA version that is unable to bind to dsDNA, we generated a mutant allele of *dnaA* that carries a mutation in the DNA-binding domain, *dnaAR387C*, which is expected to disrupt DNA binding based on studies on *E*. *coli* DnaA [24]. DnaA could be efficiently purified (Fig 3A) and showed little degradation (Fig 3B). To test for DNA binding *in vitro*, we used a 624 bp fragment containing *oriC*, to which purified wild type DnaA bound with high affinity in gel shift experiments (Fig 3C), in contrast to a 532 bp fragment lacking *dnaA* boxes (Fig 3D). Contrarily, DnaAR387C showed only non-specific binding to the *oriC* fragment (Fig 3E). To further quantify this point, we immobilized a 632 bp PCR fragment containing *oriC* on an SPR (surface plasmon resonance) chip. Wild type DnaA showed higher affinity to *oriC* DNA in the presence of ATP (Fig 3G) than in the absence (Fig 3F), whereas DnaAR387C did not show any DNA binding activity in SPR experiments irrespective of the absence or presence of ATP (Fig 3F and 3G). Interestingly, the DNA-binding mutant DnaA protein still formed foci at the replication machinery, but not at *oriC* regions (co-localization in only 0.5% of the cells, n = 200, see overlay in Fig 4A), verifying the findings that DnaA is bound to the replication forks via protein/protein interactions to YabA and to DnaN (the sliding clamp of the polymerases) [8], and to *oriC* via direct DNA binding. The accumulation of DNA-binding deficient DnaA at the replication machinery in addition to that of wild type DnaA present in the cells reveals that there are sufficient binding sites to allow for the visual accumulation of mutant DnaA. Half-time FRAP recovery of DnaAR387C was 2.5 s ± 0.4 s (Fig 4A and 4B, ME21, Table 2), and not significantly different from that of wild type DnaA (Fig 4C), confirming that DnaN-bound DnaA also has a very high turnover rate. Thus, while DnaA is bound to DnaN via YabA, rather than to DNA at *oriC*, the turnover rate is similar for replication machinery-bound DnaA compared with *oriC*-bound protein.

### YabA shows similarly high turnover as DnaA

We also determined FRAP recovery for YabA (Fig 2C and 2D). YFP-YabA expressed additionally to YabA had a half-time recovery of 2.3 ± 0.2 s (strain JDS190, Table 2), and 2.3 ± 0.2 s when expressed as sole source of YabA in the cell (strain KS173, Table 2), and thus very close to that seen for DnaA (Fig 2E). However, the immobile fraction of YabA was 20%, somewhat higher than that of DnaA (Table 2). We cannot determine if this fraction is present at *oriC* or at the replication machinery, because *oriC*-co-localized YabA is more difficult to visualize than *oriC*-bound DnaA, making localization analysis much noisier and less reliable. These data suggest that individual molecules of YabA and DnaA do not remain bound to *oriC* or to the replication forks for more than few seconds and that neither DnaA nor YabA have a significant stationary subpopulation of more than 10 or 20%, respectively, at *oriC*.

### Single molecule analysis of DnaA reveals fast molecule dynamics and short residence times

**Fig 5:**
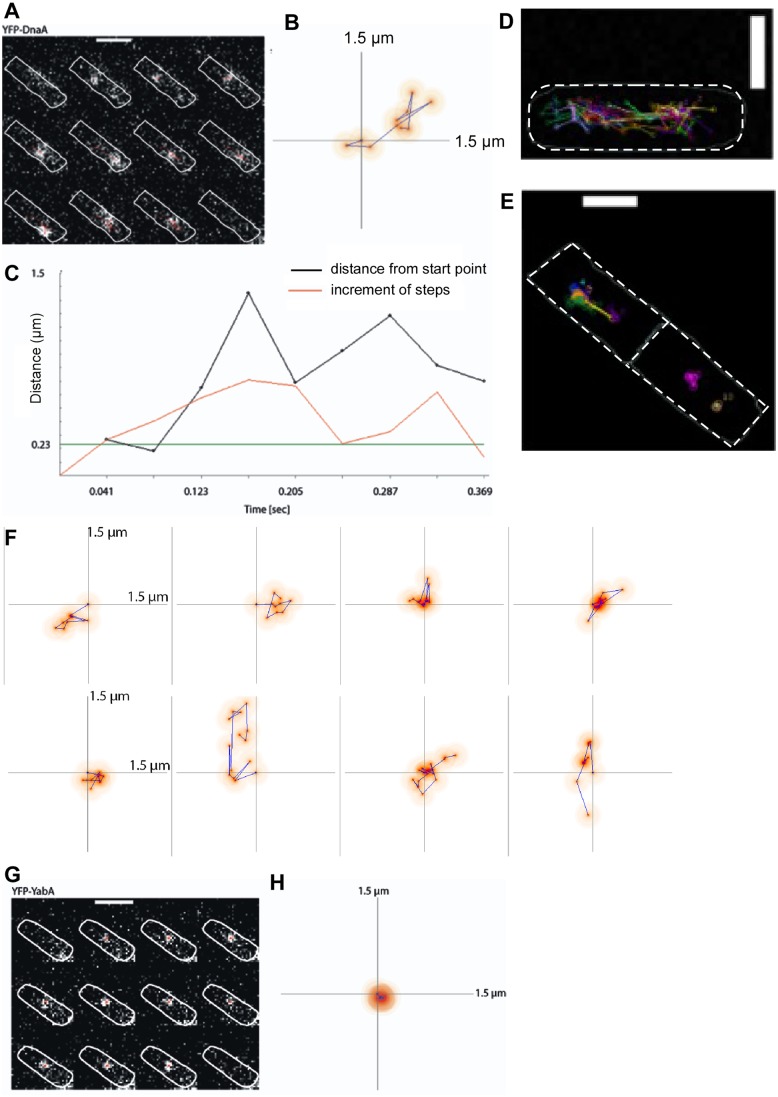
SMT experiments. A) Mobile YFP-DnaA molecule. Image sequence showing a single YFP-DnaA molecule. Each frame corresponds to a time interval of 41 ms. White line, cell outline; scale bar, 2 μm. B) Dynamic heat map of the YFP-DnaA trajectory shown in (A). Coordinate plane corresponds to a length of 1.5 μm. The beginning of the track starts at the point of origin. C) Diagram visualizing the distance from the origin (black) over time, the increment over time (red) and the defined stopping area (0.23 μm, green). D) Overlay of many tracks (each indicated by a different colour) of YFP-DnaA molecules in a single cell (outlines indicated by white line). White bar 2 μm. E) Overlay of many tracks (each indicated by a different colour) of YFP-YabA molecules in two cells (outlines indicated by white line). White bar 2 μm. F) Heat maps of YFP-DnaA showing different patterns of movement. G) Image sequence showing single YFP-YabA molecules. Each frame corresponds to a time interval of 41 ms. White dashed line, cell outline; scale bar, 2 μm. H) Dynamic heat map of the YFP-YabA single molecule trajectory shown in (G). Coordinate plane corresponds to a length of 1.5 μm. The beginning of the track starts at the point of origin.

To use a second method for analyzing DnaA dynamics in live cells, we visualized and tracked single YFP-DnaA molecules using single molecule fluorescence microscopy and single molecule tracking (SMT). To facilitate tracking of YFP-DnaA by generating fewer signals per cell, we took advantage of the fact that ectopically expressed YFP-DnaA (strain ME15) behaved similarly in FRAP experiments as YFP-DnaA expressed from the native locus (Fig 4A). We induced YFP-DnaA from the amylase locus with a very low concentration of xylose (0.01% instead of 0.5%) to generate only few molecules per cell that can be easily tracked, and continuously illuminated the cells at 514 nm with an exposure and interval time of 41 ms per frame. After bleaching most of the few chromophores in the cells, we observed movement of single YFP-DnaA molecules, which can be recognized by a characteristic single bleaching step to background noise (S5A and S5B Fig). We linked single molecule localizations in consecutive frames either manually or automatically and quantitatively analyzed the dynamics and the kinetics of movement. Fig 5A shows an example of a dynamic YFP-DnaA track, in which a single YFP-DnaA molecule moves from the center of the cell towards one pole and then back, to bleach in a single step between two frames (also see S1 and S2 Movies for exemplary tracks). The corresponding heat map (Fig 5B) shows that the molecule did not arrest at one site for more than one interval, and Fig 5C shows that the step size of movement has mostly been in increments of more than 230 nm. Molecules that move in steps of less than 230 nm are considered static (230 nm corresponds to 2.3 pixels in our setup where movement between pixels is just about detectable), e.g. shown in S5C, S5D and S5E Fig for YFP-DnaA (see also S3 Movie), or in Fig 5G and 5H for YFP-YabA. Importantly, we were able to observe molecules undergoing transitions between mobile and static events in addition to examples of purely mobile or immobile movements (Fig 5F).

For all tracks observed from the different fluorescent protein fusions (Table 3) we first calculated apparent diffusion constants (D*) from the mean square displacement (MSD). Taking into account all molecules (185 trajectories of an average length of 5.7 time points), YFP-DnaA shows an apparent diffusion rate of 0.68 μm^2^/s (Table 3), which is about an order of magnitude less than that of free GFP (7 to 14 μm^2^/s) [25, 26]. Even accounting for the fact that YFP-DnaA has a size of 78 kDa and might migrate as a dimer, the diffusion rate is low, which indicates that movement of DnaA is restrained. This is most likely generated through its non-specific interaction with DNA since it also acts as a transcription factor at several sites on the chromosome [27]. An overlay of all tracks monitored in a single cell shows that movement of DnaA occurs mostly in the middle of the cell, and rarely at the cell poles (Fig 5D), consistent with the idea that most dynamic DnaA molecules move through the nucleoid and are constrained in their diffusion through DNA-interactions. However, we cannot distinguish between constrained movement and freely diffusing DnaA molecules, which should also exist in the cells.

**Table 3:** SMT analysis.

Strain	Diffusion constant [μm^2^/sec]	SEM
ME15 (*amyE*::*yfp-dnaA*)	0.676	0.061
ME20 (*amyE*::*yfp-dnaAE183Q*)	0.983	0.070
KS167 (*amyE*::*yfp-yabA* Δ*yabA*)	0.158	0.016
DnaA-YFP^sw^ *E*. *coli* (original locus)	0.335	0.012
KS192 (*amyE*::*yfp-dnaA*, Δ*soj-spo0J*)	0.313	0.020
Spo0J-YFP (*amyE*::*spo0J-yfp*)	0.025	0.001
	average residence time [ms]	
ME15 (*amyE*::*yfp-dnaA*)	197	
ME20 (*amyE*::*yfp-dnaAE183Q*)	163	
KS167 (*amyE*::*yfp-yabA* Δ*yabA*)	259	
DnaA-YFP^sw^ *E*. *coli* (original locus)	258	
KS192 (*amyE*::*yfp-dnaA*, Δ*soj-spo0J*)	257	

**Fig 6:**
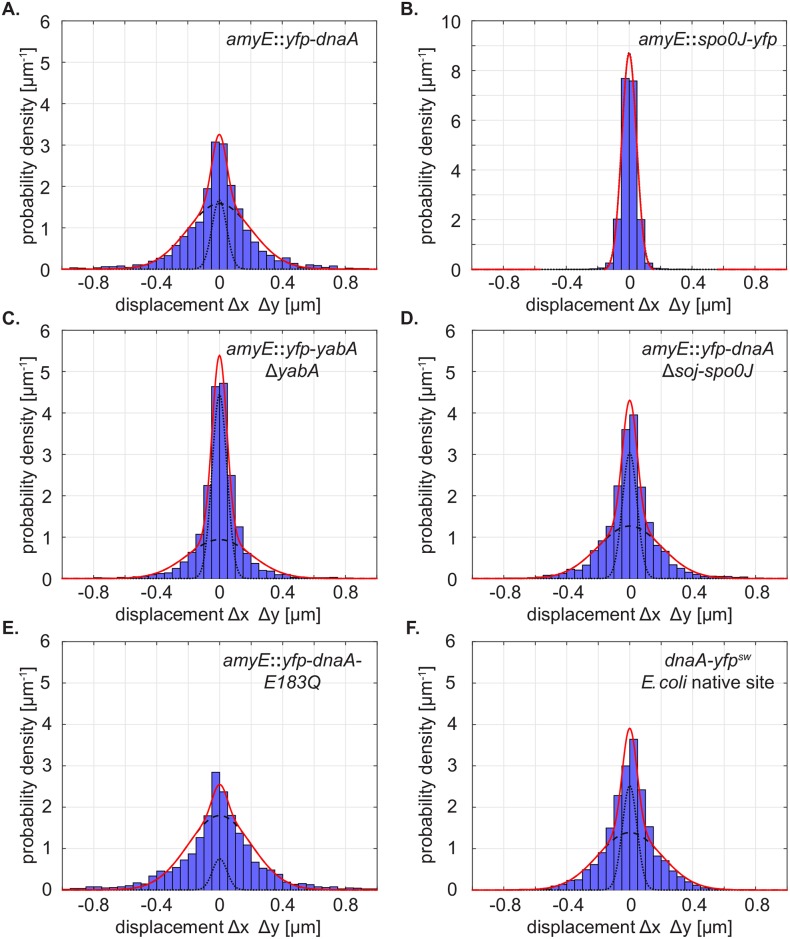
Mobility of proteins analyzed by single molecule tracking. Shown are histograms of frame-to-frame displacements in x- and y-directions. To calculate the population size and the diffusion coefficients of the individual populations, the distributions were fitted by a Gaussian mixture model. The red line represents the sum of the two Gaussian distributions, and the dotted and dashed lines represent the single Gaussian distributions that correspond to the slow and fast mode of motion. A) YFP-DnaA expressed from the ectopic *amyE* locus under the control of a xylose inducible promoter. B) YFP-Spo0J expressed from the ectopic *amyE* locus under the control of a xylose inducible promoter. C) YFP-YabA expressed from the ectopic *amyE* locus under the control of a xylose inducible promoter in a strain deleted for YabA. D) YFP-DnaA expressed from the ectopic *amyE* locus under the control of a xylose inducible promoter in a strain lacking Soj-Spo0J. E) YFP-DnaAE183Q expressed from the ectopic *amyE* locus under the control of a xylose inducible promoter. F) *E*. *coli* DnaA-YFP^sw^ expressed from the native locus. All fluorescent protein fusions were tracked at time intervals of 41 ms. See Table 4 for diffusion coefficients and the size of mobile and immobile populations.

We also analyzed the step size distribution of all the fluorescent protein fusions to identify subpopulations of movement (Fig 6). Particles undergoing Brownian motion move in increments that follow a normal (Gaussian) distribution with mean zero (the longer the displacement, the further away is the value from “0”) and a variance σ^2^, which scales with the diffusion coefficient. Thus, the higher the variance of the distribution, the higher is the diffusion coefficient of the particle. In case of YFP-DnaA, the shape of the histogram could not be described well by a single Gaussian distribution (S6A Fig). So we applied a multivariate Gaussian distribution fit assuming two modes of motion (Fig 6A), which also reflects our previous observations (Fig 5A and 5B and S5C and S5D Fig). Thereby, we determined a static/slow moving fraction (dotted line, Fig 6A), with an apparent diffusion constant of 0.027 μm^2^/s (Table 4), and a dynamic fraction (dashed line, Fig 6A) with D* = 0.51 μm^2^/s. From the area underneath the two curves, we can determine that 20% of DnaA molecules are static and 80% are dynamic molecules revealing that most DnaA molecules are moving in search for binding sites on the chromosome. This more discriminative analysis of DnaA movement reveals two distinct populations, and together with the heat maps shows that DnaA can change between static and dynamic movement within short periods of time (few hundred milliseconds).

**Table 4:** Determination of mobile and static fractions(simultaneous fit over all step size distributions).

strain	D* [μm^2^ s^-1^]	Fractions [%] static/mobile
ME15 (*amyE*::*yfp-dnaA*)	0.027^1^/ 0.51^2^	20^1^ / 80^2^
KS167 (*amyE*::*yfp-yabA* Δ*yabA*)		52^1^ / 48^2^
KS192 (*amyE*::*yfp-dnaA*, Δ*soj-spo0J*)		36^1^ / 64^2^
ME20 (*amyE*::*yfp-dnaAE183Q*)		9^1^ / 91^2^
DnaA-YFP^sw^ *E*. *coli* (original locus)		30^1^ / 70^2^
Spo0J-YFP (*amyE*::*spo0J-yfp*)		100^1^ / 0^2^

1: static fraction 2: dynamic fraction

DnaA was surprisingly dynamic so as a control we wished to analyze a protein presumably diffusing much slower. Therefore, we tracked Spo0J-GFP (1104 molecules, average track length 8.5 time points), which binds to at least 10 specific sites surrounding the origin regions on the chromosome [28]. In agreement with slow recovery in FRAP experiments [29], we found that Spo0J molecules are largely static (Fig 6B, Table 4), likely representing molecules sequestering *parS* sites. Similarly, heat maps of Spo0J-YFP show entirely static molecules (S9B Fig).

Additionally, we performed two control experiments to rule out that we are not missing a dynamic fraction of DnaA under our conditions. First, we tracked YFP-DnaA expressed from the original gene locus as sole source of the protein. We induced with 0.1% xylose, which leads to lower cellular levels of DnaA (S1A Fig), and tracked the movement using faster (10 ms) stream acquisition settings. YFP-DnaA showed a very similar step size distribution under this condition compared to the low-induced ectopically expressed YFP-DnaA tracked in time intervals of 41 ms (S6B Fig). We found that 19% of the steps showed static movement (D* = 0.2 μm^2^/s), and 81% of the steps corresponded to dynamic movement. Further analysis of the square displacement distribution using a three-component analysis indicated that we are unlikely to miss a highly mobile fraction of YFP-DnaA (S6C Fig). Second, we used our setup to track mNeongreen (a variant of LanYFP from *Branchiostoma lanceolatum*, distantly related to GFP [30]) in *E*. *coli* cells, using fast stream acquisition with an exposure time and interval time of 4 ms (S4 Movie). An overlay of mNeongreen tracks obtained in two cells, reveals that it diffuses throughout the cells (S7A Fig), in contrast to the more centrally restrained movement of YFP-DnaA (Fig 5D). The motion can be described by a single population fit referring to dynamic molecules, which diffuse with D* = 3.3 μm^2^/s, much faster than the dynamic fraction of YFP-DnaA with D* = 0.51 μm^2^/s (S7B Fig). These experiments show that our experimental setup is able to track slowly and quickly diffusing molecules.

**Fig 7:**
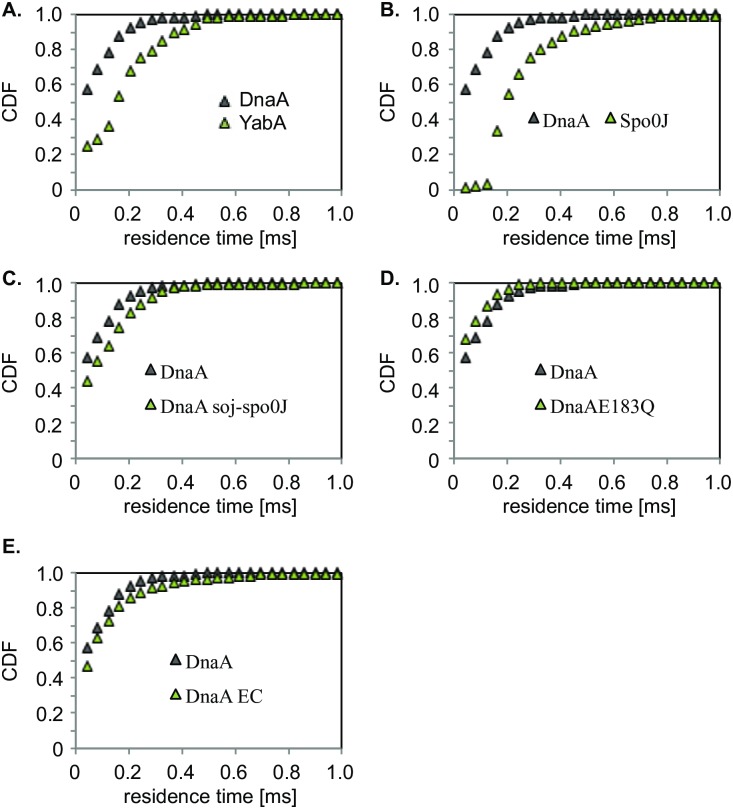
Cumulative distribution functions (CDF) of residence times. The plots show the cumulative probability that a molecule remains in a circle of radius 230 nm for the indicated time. A) Comparison of YFP-DnaA expressed from the ectopic *amyE* locus under the control of a xylose inducible promoter (ME15) and YFP-YabA expressed from the ectopic *amyE* locus under the control of a xylose inducible promoter in a strain deleted for YabA. B) Comparison of ME15 and YFP-Spo0J expressed from the ectopic *amyE* locus under the control of a xylose inducible promoter C) Comparison of ME15 and YFP-DnaA expressed from the ectopic *amyE* locus under the control of a xylose inducible promoter in a strain lacking Soj-Spo0J. D) YFP-DnaA (ME15) compared with YFP-DnaAE183Q expressed from the ectopic *amyE* locus under the control of a xylose inducible promoter. E) YFP-DnaA (ME15) compared with *E*. *coli* DnaA-YFP^sw^ expressed from the native locus. In all comparisons, the null hypothesis that the data of the curves come from the same distributions is rejected at the 5% significance level (two-sample Kolmogorov-Smirnov (KS) -test).

We wished to determine the average residence time of YFP-DnaA on all sites on the chromosome. Average residence time was computed by considering the intervals, both forward and backward in time, where a track stays within a radius of 230 nm. Hence, each track is decomposed into a set of non-overlapping time intervals. To remove the contribution of slowly diffusing mobile molecules, we consider only static intervals of length greater than or equal to three frames. For YFP-DnaA, and given our track length distribution, we estimated that the probability of a free molecule being erroneously counted as static is 9%. S8 Fig shows a histogram of the percentage of molecules stopping for 3, 4, 5, 6 and 15 time intervals. From all stopping events, the cumulated periods of residence for YFP-DnaA are shown in Fig 7A, from which we calculated an average residence time of 197 ms (Table 3). This number from SMT is much lower than the turnover at *oriC* determined by FRAP experiments because this approach measures transient binding events at all places on the chromosome besides *oriC* (e.g. promoters having *dnaA* boxes plus non-specific DNA binding), or at the replication machinery. The small relative number of YFP-DnaA molecules bound at *oriC* or at the replication machinery compared to the dynamic molecules also contributes to this disparity. Nonetheless, we can conclude that single DnaA molecules stay transiently at individual sites for about 200 ms on average, revealing extremely rapid turnover on its chromosomal binding sites.

### YabA and Spo0J show more static movement than DnaA

In contrast to YFP-DnaA, few mobile YFP-YabA molecules were observed, and mostly static spots appeared and disappeared. The fact that YabA was mostly static can be seen from the overlay of tracks in a single cell (Fig 5E), which is strikingly different from that of DnaA (Fig 5D), and in agreement with the idea that YabA binds mostly to the centrally located replication machinery, or to origin regions positioned around the quarter sites in the cell, with the measured recovery half-time of 2.5 s (Fig 2C). Consequently, the apparent diffusion coefficient of YFP-YabA is much lower than that of DnaA with D* = 0.16 μm^2^/s as calculated by MSD analysis (Table 3, 171 molecules, average track length 8.5 time points) and the step size distribution (Fig 6C) shows a much higher static fraction of YabA compared with that of DnaA (Fig 6A). 52% of YabA molcules were static, and 48% were mobile (Fig 6C, Table 4). YFP-YabA exhibited similar residence times as Spo0J-YFP (259 and 275 ms, Table 3) and resided for longer times in a radius of 230 nm compared to YFP-DnaA molecules. When we restricted the dwell time analysis to tracks that were at least 10 frames long, we found that YFP-YabA and Spo0J-YFP stayed about 2.5 and 5 frames longer respectively in the same radius than YFP-DnaA (S1 Table and S10A and S10B Fig). These data show that YabA does not diffuse through the nucleoid, neither in a complex with DnaA, nor alone in a constrained motion like DnaA, because a rapidly diffusing fraction similar to that of DnaA is not observed (while a YabA-YFP tetramer has about the same size as a YFP-DnaA dimer). Rather, YabA arrests at sites where likely DnaA and/or DnaN are present, and dissociates off to move freely through the cell. YabA could therefore act as a “chaser” for DNA-bound DnaA, leading to the dissociation of DnaA assemblies, possibly together with Soj. Note that a similar experimental setup showed that different regions on the chromosome do not move at this time scale [31], such that the mobile molecules detected for DnaA and for YabA do indeed move between different sites on the chromosome rather than the chromosome moving with DnaA, or through the entire cell for YabA.

### Deletions in *yabA* or *soj* increase half time turnover of DnaA at *oriC*

ADP-bound Soj has been shown to negatively affect the multimerization of DnaA [14], which is assumed to be an important step in replication initiation. Also, YabA has been shown to decrease the co-operativity of DnaA binding to DNA *in vitro* [10]. The deletion of *soj* leads to a very modest over-initiation phenotype (1.3 times more origins than wild type cells [16]), while a *yabA* deletion leads to a slower growth rate and a 1.7 fold increase in *oriC* number [8]. We wondered if a loss of any of the negative regulators of DnaA would have an effect on the dwell time of DnaA at *oriC*. Indeed, half-time recovery was extended to 3.8 ± 0.3 s in the absence of YabA (Fig 2F and 2G, strain KS171 Table 2), and to 4.3 ± 0.4 s in cells devoid of Soj/Spo0J (Fig 2I and 2J, strain KS175, Table 2). Note that *yabA* mutant cells have more background signal for YFP-DnaA (Fig 2F), i.e. contain more diffusive YFP-DnaA molecules, because DnaA is no longer tethered to the replication forks [9]. Statistical analysis shows that the recovery of YFP-DnaA in the absence of YabA is significantly lower than in wild type cells (Fig 2H), with a p-value of 0.002. Absence of Soj/Spo0J is also statistically highly relevant (Fig 2K), with p = 0.001.

These data strongly suggest that both YabA and Soj act as chasers of DnaA at *oriC*. Consistent with this idea, when we deleted *soj/spo0J*, YFP-DnaA molecules became more stationary, which can be seen in the more narrow step size distribution (Fig 6D; 36% static and 64% dynamic, compared to 20/80% for wild type cells, Table 4), and showed increased residence times (Fig 7C and S8B Fig) in SMT experiments. From the resting times of 3 or more frames, we calculated average residence time of 197 ms for YFP-DnaA in wild type cells, and 257 ms in *soj/spo0J* mutant cells (KS92, 125 molecules, average track length 6.6 time points, Table 3). For tracks longer than 10 frames, the difference was no longer detectable showing that the loss of *soj/spo0J* affects only short residence times (S1 Table and S10C Fig). Taken together it is apparent that in the absence of Soj/Spo0J, individual DnaA molecules transiently arrest for longer periods of time at their chromosomal binding sites, and not only at *oriC*. It was difficult to track YFP-DnaA in the absence of YabA, for a reason that is unclear to us, so we could not determine the effect of the absence of YabA on single molecule movement of DnaA.

### A DnaA mutant impaired in ATPase activity decreases residence times and leads to underinitiation of replication

**Fig 8:**
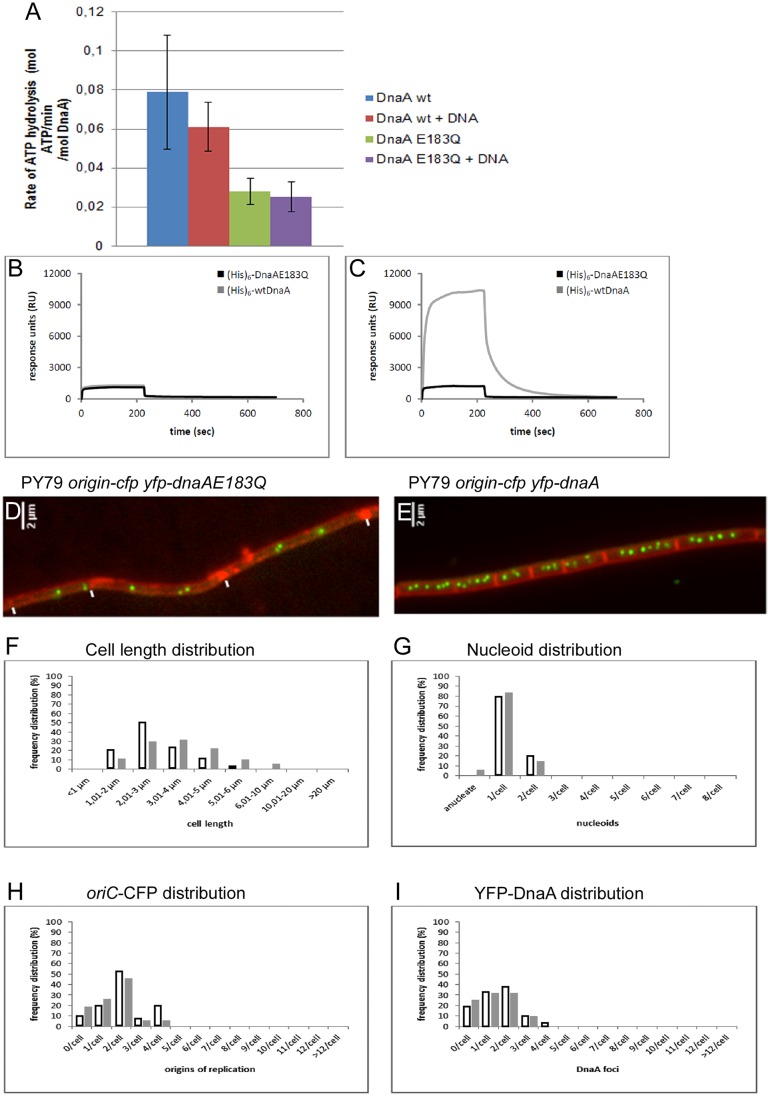
DnaAE183Q mutant shows reduced ATPase activity and an underinitiating phenotype. A) Coupled ATPase assay of purified wild type DnaA or of DnaAE183Q (10 μM). B) Surface plasmon resonance [SPR] experiments with DnaA and DnaAE183Q [2.5 μM] binding to *oriC*-DNA [0.25 pmol]. Black line: DnaAE183Q, grey line: wtDnaA. C) SPR in the presence of 2.5 mM ATP. D-E) Fluorescence microscopy analysis of cells growing exponentially in rich (LB) medium, D) expressing YFP-DnaAE183Q for 60 minutes, E) expressing YFP-DnaA for 60 minutes. Note that cells contain more *oriC* regions in rich medium compared with minimal medium as in Figs 1 and 2. The effect of induction of mutant DnaA is more apparent in rich medium. Septa are indicated by white lines, and are more difficult to see after induction of mutant DnaA. F)-I) Increased cell size and reduced number of origins in exponentially growing cells expressing DnaAE183Q for 60 min in minimal medium, white bars wild type cells, grey bars cells after induction of DnaAE183Q, F) Diagram showing the distribution of cell length, bars show percentage of cells with a certain size, white bars wild type cells, G) Diagram showing percentage of cells containing one or two nucleoids, or none, H) Diagram showing the percentage of cells containing a certain number of origins, I) Diagram showing the percentage of cells containing no YFP-DnaA foci, or different numbers of foci.

The above data suggest that an increase in the residence time of DnaA at *oriC* leads to over-initiation of replication and that both YabA and Soj act to decrease the residence time of DnaA, both at longer residency-time targets, i.e. the initiation complex at *oriC* and the replication forks, and at shorter-bound targets on the chromosome. If this were true, then a reduced average residence time of DnaA should lead to under-initiation. To test this notion, we generated a mutant DnaA protein that has strongly reduced ATPase activity (Fig 8A), DnaAE183Q. We reasoned that this mutant protein might be compromised for initiation activity. Purified DnaAE183Q bound to *oriC* DNA with similar affinity as wt DnaA (Fig 8B), but did not show an increase in binding activity after addition of ATP as is true in the case for wild type DnaA (Fig 8C). Expression of *DnaAE183Q* from an ectopic site on the chromosome and in the presence of wild-type *dnaA* resulted in the elongation of cells, and the number of origins per cell decreased considerably compared to that in wild type cells, which was especially evident in rich medium, where wild type cells have between 4 to 8 origins, whereas 60 minutes after induction of mutant DnaA, cells contained between 2 and 4 origins (Fig 8D and 8E). When we investigated the expression of the mutant allele in minimal medium, cells also became considerably longer that wild type cells (Fig 8F), had a decreased number of nucleoids (Fig 8G) and of origins (Fig 8H), and also showed fewer YFP-DnaA foci per cell (Fig 8I). These data show that the mutant protein acts in a dominant negative way and leads to under-replication. Interestingly, tracking of YFP-DnaAE183Q revealed that the protein arrests less frequently than the wild type protein (average residence time 163 ms, strain ME20, Table 3, Fig 7D, S10D Fig). The difference in residence time is even more apparent when only tracks longer than 10 time points are used for the calculation of average residence time: here, wt DnaA has a residence time of 342 ms, while underinitiating mutant DnaA has 205 ms (S1 Table). Consequently, DnaAE183Q (144 tracks with an average length of 5.7 time points) showed a higher apparent diffusion coefficient 0.98 μm^2^/s than wild type YFP-DnaA (0.68 μm^2^/s) (Table 3) as calculated using MSD analysis, and which is also apparent from the increase in the fraction of the mobile molecules (91% versus 80% for wtDnaA) and a decrease in the static fraction (9 versus 20%) (Fig 6E and Table 4). These findings suggest that a decrease in average residence time of DnaA on the chromosome leads to a reduction in initiation frequency.

### DnaA oscillates between origin regions in *E*. *coli*

**Fig 9:**
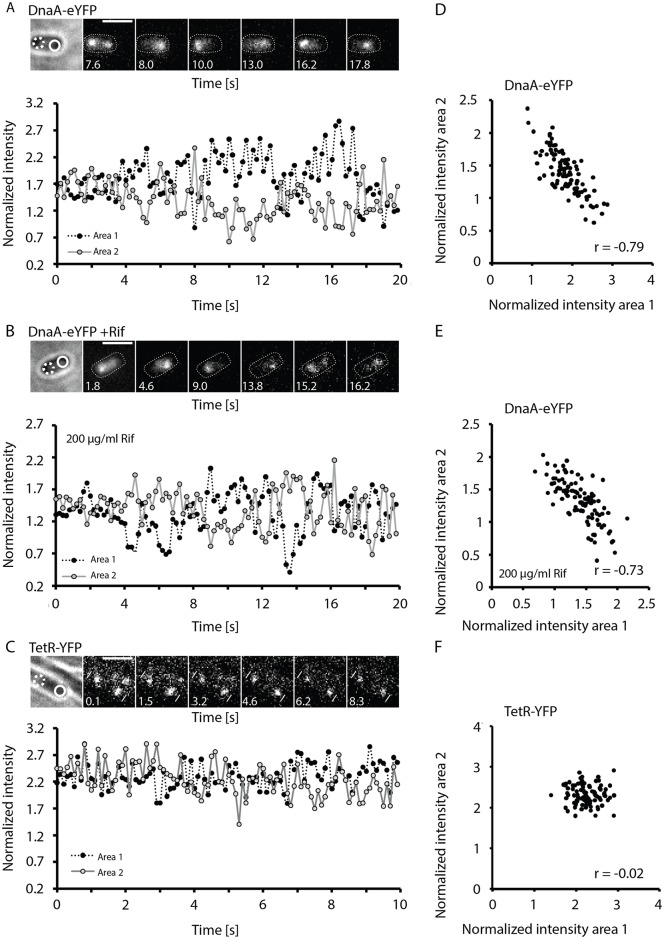
Oscillation of DnaA-YFP^sw^ in *E*. *coli*. Fluorescence microscopy images of *E*. *coli* cells, A) expressing DnaA-YFP^sw^ during exponential growth, B) 60 min after the addition of 200 μg/ml rifampicin, C) *B*. *subtilis* cells expressing TetR-YFP binding to a *tetO* array near *oriC*. Fluorescence intensities of two areas, each containing one fluorescence signal, were measured and normalized to the total fluorescence intensity of the cell. Intensities of both areas were plotted over time. Area 1 (black) and area 2 (white). Image sequences displaying the oscillation of DnaA-YFP^sw^ (A–B) and the static TetR-YFP (C) are shown. White lines, cell borders; scale bars, 2 μm; white dashed lines, cell outlines; black open circle, area 1; green open circle; area 2. To visualize the correlation between the two measured areas they were plotted against each other (D–F). Correlation coefficients (r) were determined.

A functional DnaA-YFP sandwich (^sw^) fusion has been shown to localize as foci in *E*. *coli*, either at *oriC* regions, or also at the DAT locus, which contains many *dnaA* boxes [17]. In another study, DnaA was reported to form foci along the cell membrane, in a helical pattern [18]. Of note, epifluorescence laser illumination (stream, exposure time = 200 ms) was used, such that only many fluorescent proteins being present at a certain position will be seen as a spot, rather than single FP molecules as in SMT. Intriguingly, DnaA-YFP^sw^ was highly mobile, fluorescent signals were clearly seen to move along the chromosome between the cell halves (S5 Movie and Fig 9A). We quantified the intensity of fluorescent patches in the cell halves, relative to each other and found that DnaA oscillates between opposite cell halves in a time frame of few seconds. The plot shows that an increase in intensity in one cell half is accompanied by a correlated decrease in the other half. When the normalized intensity in one half is plotted against that in the other half (Fig 9A, right panel), a clearly inverse correlation is apparent. When the signal of a *tetO* array bound by TetR-YFP is analysed under identical conditions (Fig 9C), no correlation between signals in the two cell halves is observed (Fig 9C, right panel). We wondered whether the removal of DnaA from one part of the chromosome (including *oriC*) might be driven by RNA polymerase engaged in transcription. We therefore performed experiments in cells that were treated with rifampicin for 60 minutes. Fig 9B shows that DnaA-YFP^sw^ still oscillated between chromosome regions, without any noticeably altered timing, and still showed a highly inverse correlation between normalized intensities (Fig 9B, right panel). These data suggest that coordinated binding and unbinding of DnaA also occurs on a time scale of a few seconds in *E*. *coli*, independent of transcription. Interestingly, DnaA oscillation is much faster than that of MinD, a membrane-associated regulator of cell division, which oscillates between the cell poles in a frame of about 20 seconds, driven by the MinE “chaser” [32, 33]. It will be highly interesting to investigate the involvement of ATP in this process and to identify the chaser of *E*. *coli* DnaA. In SMT experiments, *E*. *coli* DnaA showed rapid movement (S6 Movie), however, diffusion rates were slower (0.34 μm^2^/s) and residence time longer (258 ms, Fig 7E) than those of *B*. *subtilis* DnaA (Table 3, 279 molecules, average track length 9.6 frames). Like BsDnaA, EsDnaA showed patterns of static and solely dynamic movement, as well as changes between movement and resting periods (S9A Fig). Additionally, 70% of EcDnaA were dynamic (Table 3, Fig 6F), compared with 80% for BsDnaA, revealing that the protein is highly mobile, but somewhat less dynamic than its Gram positive counterpart. Therefore, DnaA clearly has very fast binding and unbinding dynamics in two model bacteria, and possibly in many other bacteria.

## Discussion

It has been elegantly shown that DnaA binds to several high affinity binding sites within *oriC*, and can form a helical superstructure upon spreading to further sites at *oriC*, leading to strand opening and to the recruitment of DNA helicase loaders [15, 34–36]. Our data show that indeed, DnaA is visibly present at *oriC* regions throughout the *Bacillus* cell cycle, however, its average residence time is surprisingly short, around 2.5 s. In *E*. *coli*, the observed oscillatory dynamics also suggest a residence time at *oriC* in the lower seconds range. Evidently, the cell ensures at a time frame of seconds that an *oriC*-bound DnaA supercomplex can not build up prematurely. A fast binding turnover is clearly helpful if not crucial for avoiding the formation of DnaA multimers at *oriC*, whose stable generation would lead to initiation. Additionally, while over-initiation is detrimental or even fatal for cells, high DnaA turnover may be advantageous when cells have to adapt to environmental changes, and may allow rapid stabilization of DnaA at *oriC* and thereby rapidly trigger initiation or allow for an increase in initiation frequency when needed. It should be noted that we have studied the dynamics of DnaA in bulk culture, so residence times may vary considerable within the cell cycle, and it is likely that prior to initiation of replication, DnaA exchange at *oriC* is strongly decreased, leading to spreading into the DnaA-trio sequences adjacent to the *dnaA* boxes [37].

We studied the localization of two mutant alleles of DnaA that were generated based on studies performed on *E*. *coli* DnaA. Mutation E204Q (*B*. *subtilis*: E183Q) leads to reduced ATPase activity [38, 39], while mutation R407C (*B*. *subtilis*: R387C) abolished dsDNA binding [40]. We show that the corresponding mutations in *B*. *subtilis* have the same effect, and ectopic induction of DnaAE183Q leads to a dominant negative effect in initiation activity, showing that reduced ATPase activity interferes with the action of DnaA even if wild type protein is present. Purified DnaAE183Q still bound to *oriC* DNA like wild type DnaA, but affinity could not be stimulated by the addition of ATP, as is the case for wild type protein, showing that ATPase activity affects high affinity binding of DnaA. YFP-DnaAE183Q formed fewer fluorescent foci in the cells and led to a strong underinitiation phenotype. Conversely, non-DNA binding YFP-DnaAR387C did not affect initiation frequency, and still visibly assembled at replication forks but not at origin regions. These data reinforce the idea that DnaA binds to the replication machinery via the YabA adaptor protein and DnaN, and to origin regions via direct DNA binding. Mutant DnaA accumulated at the replication forks in the presence of wild type DnaA, showing that there are ample binding sites, and that neither DnaN nor YabA are limiting factors to attach additional DnaA molecules, in support of the idea that DnaN acts as a sink for DnaA to titrate a considerable amount of the inducer away from duplicated and separated origin regions.

While in *B*. *subtilis*, based on our data, molecules at *oriC* exchange between the replication forks, origins and other sites on the chromosome in a seemingly stochastic manner, in *E*. *coli*, an oscillation between two cell halves (and thus most likely between origins) can be observed. The mechanism of this coordinated association and dissociation is unknown, but may involve a “chaser” molecule, in analogy to MinE leading to MinD disassembly at a cell pole [41, 42]. It will be highly interesting to elucidate how this oscillation is driven at a molecular level. In any event, DnaA shows rapid turnover of molecules on the chromosome in two model bacteria compared with a residence time of 4 min for *lac* repressor being bound to *lacO* [20], 2 to 2.5 minutes for *tetO*-bound TetR [21, 22], 2.5 minutes for LacI-GFP binding to a *lacO* array (this work), or 2 minutes for the SMC protein remaining within condensation centres located near the origin regions on the chromosome [29].

Interestingly, the ParB partner of Soj (ParA), the Spo0J protein, shows very static behavior. Compared with DnaA, we could not observe any exchange between bound molecules surrounding origin regions and possible free molecules. The number of free Spo0J-YFP found in this study is likely an underestimate of the actual number, because Spo0J-YFP was tracked at 41 ms for comparison to DnaA dynamics, so in this case fast diffusing molecules may have been lost, but clearly, binding of Spo0J is highly static, such that it is able to generate long range connections within the origin region, as has been seen in Hi-C analyses [43], over long periods of time.

Importantly, an increase in residence time through the deletion of DnaA regulators YabA and Soj (ParA) in *B*. *subtilis*, leads to over-initiation, showing that rapid turnover at *oriC* is modulated by two interactor proteins, both of which increase the rate of dissociation of DnaA, in agreement with findings that YabA and Soj interfere with cooperative binding of DnaA or with multimerization, respectively, *in vitro* [10, 44]. Therefore, YabA confers a dual role, acting as a tether of DnaA to the replication machinery, and also as a chaser of DnaA binding at *oriC* [10, 11]. Mutant DnaA protein that has lost DNA-binding activity is no longer able to bind to *oriC*, but is still sequestered to the replication machinery, verifying the idea that a considerable number of DnaA molecules are bound at the forks, and—although rapidly exchanged—are not available for *oriC* binding, until replication has terminated. Additionally, a decrease in DnaA ATPase activity (E183Q mutation) leads to decreased binding to *oriC* and other sites on the chromosome, showing that this aspect of DnaA activities also serves as an additional layer of replication control in *B*. *subtilis*.

Our data suggest that binding of many DnaA molecules to *oriC* visibly occurs at many times in the cell cycle, and that one major function of YabA and of Soj is to counteract this event in order to prevent over-initiation. However, YabA clearly has a second function in recruiting DnaA to the active replication machinery. The fact that generally, YabA can be seen to accumulate at the replication machinery, rather than at *oriC*, yet interacts with origin regions throughout the cell cycle, suggests that there are more binding sites at the forks than at *oriC*, which is also true for DnaA. This is corroborated with recent experiments showing that DnaN dimers remain bound to the lagging strand for an extended time, and are thus deposited behind the replication forks [45, 46]. Thus, DnaN provides many binding sites for YabA and DnaA, for which DnaA evidently has similar association/dissociation kinetics. Therefore, DnaA moves between *oriC*, different sites on the chromosome, and the replication machinery, with the latter providing many binding sites to act as a *datA*-like sink for DnaA binding. Together, DnaN binding via YabA, inhibition of cooperative DNA binding of DnaA at *oriC* by YabA, and inhibition of multimerization, which would lead to strand opening at *oriC*, by Soj provide efficient means to restrict initiation activity to once per cell cycle.

SMT experiments show that DnaA has very short residence times in *B*. *subtilis* as well as in *E*. *coli*, in the range of few hundred milliseconds. It should be noted that the values of 200 or 260 ms, respectively, determined in our study are underestimates of the true dwell time at binding sites due to interference by photobleaching, i.e. bleaching of molecules that are bound on the DNA. However, the actual values in the cell will not be strikingly different. SMT is therefore a powerful technique to determine *in vivo* protein turnover rates at loci that appear to consist of static foci using epifluorescence microscopy, which has also been shown for e.g. proteins involved in DNA mismatch repair [47] or in chromosome segregation [48]. Our work reveals that two bacterial species from different phyla avoid long residence times of the initiator protein and employ tight turnover rates to ensure tight initiation control throughout the cell cycle.

## Materials and methods

### Growth of cells

All cells imaged in this study were growing exponentially, and in a non-synchronized manner. Average dwell times (exchange rates) may be different during certain periods of the cell cycle, e.g. for YFP-DnaA during initiation of replication. For most of the cell cycle, initiation activity of DnaA is repressed to prevent reinitiation events, and therefore, the data determined by FRAP and SMT analysis predominantly reflect the dynamics of DnaA during its initiation-repressed state of activity.

### Construction of strains

In order to study the localization behavior of YFP-YabA with respect to the origin of replication and the replication machinery, a strain with a CFP-tagged origin and an mCherry-tagged replication machinery was generated. To combine the CFP tagged origin with YFP-YabA, competent cells of PG208 (*spo0J*::*lacO cassette*, *thrC*::*lacI-cfp*) were transformed with chromosomal DNA of JDS190 (*amyE*::*P*_*xyl*_*yfp-yabA*, *ΔyabA*), which gave rise to strain KS119. The chloramphenicol resistance in PG1159 (*dnaX-mcherry*) was changed to tetracylcline resistance, resulting in strain KS114. Cells of KS119 were transformed with chromosomal DNA of KS114 (*dnaX-mcherry*) to obtain KS140 (*origin-cfp*, *amyE*::*yfp-yabA*, *ΔyabA*, *dnaX-mcherry*). To analyze YFP-DnaA in a Δ*yabA* background, KS171 was generated transforming competent PG1037 cells (*P*_*xyl*_ *yfp-dnaA*) with chromosomal DNA of KS173 (*ΔyabA*::*phleo amyE*::*yfp-yabA*). To test for successful *yabA* deletion a test-PCR was performed. To study YFP-DnaA in a Δ*soj-spo0J* background, PG1037 cells were transformed with chromosomal DNA of AG1505 (*Δsoj-spo0J*::*spec*), giving rise to KS175 (*Δsoj-spo0J*::*spec P*_*xyl*_ *yfp-dnaA*).

For single molecule microscopy a strain with an ectopic copy of *yfp-dnaA* in a Δ*soj-spo0J* background was generated. To this end AHV2 (*amyE*::*P*_*xyl*_*yfp-dnaA*) cells were transformed with chromosomal DNA of AG1505 (*Δsoj-spo0J*::*spec*) resulting in KS192 (*Δsoj-spo0J*::*spec amyE*::*yfp-dnaA*). To construct a strain with YFP-YabA under an IPTG (isopropyl β- D -1-thiogalactopyranoside) inducible promoter, *yfp-yabA* was amplified from chromosomal DNA of JDS190 using primer 1036 5’-ctg gct agc aga aag gag att cct agg atg and 4737 5’-cat gca tgc cta ttt ttt att taa gaa tga cag and cloned into pDR111 via *Nhe*I and *Sph*I restriction sites (KS142). Competent wild type cells were transformed using *pDR111-yfp-yabA* to generate KS149. Competent KS183 cells (*yabA*::*phleo*) were transformed with the plasmid pKS142 (*Pspac-yfp-yabA*) or chromosomal DNA of JDS190 (*amyE*::*yfp-yabA*) to give rise to KS173 and KS167, respectively.

To visualize *yfp-spo0J*, we generated a strain that expresses the fluorescent protein fusion protein from the *amyE* locus under control of the inducible xylose promoter, We amplified the gene using primers spo0J_apa_up (5`-CAT GGG CCC ATG GCT AAA GGC CTT GGA-3`), spo0J_eco_dn (5`-CAT GAA TTC TGA TTC TCG TTC AGA CAA AAG -3`) and chromosomal DNA of *B*. *subtilis* strain PY79. We ligated the PCR product into similarly digested plasmid pSG1193 and transformed it into competent PY79 cells. For tracking of single mNeonGreen molecules, we constructed a strain carrying mNeonGreen under control of an IPTG-inducible promoter. The gene was amplified from a plasmid carrying an *E*. *coli* adapted mNeonGreen allele (kind gift of the group of Kristina Jonas) using primers mNGreen_EC_FW (5`-CACCGGTGGCGGCGGTTCTATG-3`) and mNGreen_RV (5-TAAGCATGCTTACTTGTACAGCTCGTCCATGC-3`). The gene was first inserted into the pENTR-D-Topo vector and then transferred to pHGWA using the Gateway cloning system. All strains are listed in Table 1.

### Protein purification

Protein purification was performed in two consecutive steps. The purification of (His)_6_-wild type/mutant DnaA initially began with affinity chromatography using an ÄKTA Prime apparatus (GE Healthcare) and Nickel-Sepharose columns (HisTrap HP 1 ml, GE Healthcare) and was continued by size-exclusion chromatography using an ÄKTA FPLC apparatus (GE Healthcare) and a gel filtration column (Superdex 200 10/300 GL, GE Healthcare). Prior to purification, the respective proteins were overexpressed in *E*. *coli* Rosetta (DE3) pLysS cells carrying a pETDuet-1 vector (Novagen) with an (indirectly) IPTG-inducible T7 promoter, six encoded histidines and the full gene sequence of the *dnaA* variants. Transformants were grown under vigorous shaking in LB-medium at 37°C to exponential phase (OD_600_ 0.8) and induced for 60 minutes with 1 mM IPTG. Subsequently, the cells were centrifuged (20 minutes, 4°C, 5000 rpm) and the pellet was resuspended in HEPES A (50 mM HEPES, 300 mM NaCl, pH 7.5). To prevent protein degradation a protease inhibitor was added (Complete, Roche). Afterwards, the cells were French-pressed (AMINCO French Press, Laurier Research Instrumentation) in two consecutive cycles at approximately 20000 psi and the lysate was centrifuged (30 minutes, 4°C, 16000 rpm). The clear supernatant was passed through a filter (pore-size 0.45 μm, Filtropur S, Sarstedt) before injection into the loop of the ÄKTA Prime apparatus (preequilibrated with HEPES A and HEPES B [50 mM HEPES, 300 mM NaCl, 500 mM imidazole, pH 7.5]). The proteins were loaded onto the Nickel-Sepharose column, the column was washed with 20% HEPES B and the protein eluted with 100% HEPES B in fractions of 1 ml and checked by SDS-PAGE. Fractions containing significant amounts of the desired protein were assembled and loaded onto size exclusion chromatography columns (preequilibrated with HEPES A). The peak fractions were analyzed by SDS-PAGE and only pure protein fractions were assembled and stored at 4°C.

### ATPase assays

ATPase activity was measured by using a coupled spectrophotometric pyruvate kinase/lactate dehydrogenase assay as previously described [49]. The reaction was monitored at 340 nm for 30 minutes at room temperature.

### Electromobility shift assays (EMSA)

EMSA were performed with increasing amounts (0–80 pmol) of (His)_6_-wild type/mutant DnaA and either DnaA-box containing *oriC*-DNA (0.9 pmol, linear DNA-fragment, 624 nt) or control DNA without DnaA-boxes (0.9 pmol, linear DNA-fragment, 532 nt,) under ATP-containing (2.5 mM) and ATP-free conditions. The reaction mixture with a final volume of 20 μl (27.5 mM HEPES (pH 7.6), 0.25 mM EDTA, 1.25 mM magnesium acetate, 2.5% glycerol (v/v), 0.025 mg/ml BSA, 135 mM NaCl) was incubated for 30 minutes at room temperature. Subsequently, the protein-DNA samples were mixed with 6xDNA loading buffer (30% glycerol (v/v), 300 mM boric acid, 300 mM Tris, 0.5 mg/ml bromphenol blue) and run on native polyacrylamide gradient gels (4–12%, Anamed) in 50 mM boric acid and 50 mM Tris at a constant voltage (constant 200 V, 2 hours, Power source, VWR). Afterwards, the gel was placed in a beaker containing running buffer and DNA-stain (dilution 1:60000, GelRed nucleic acid gel stain, Biotium) and rotated for 20 minutes at room temperature prior to DNA-visualization by ultraviolet light (UV Transilluminator, UVP). The values for the apparent binding constant *K*_*app*_, referred to as the protein concentration at which half of the total amount of linear DNA in the reaction is bound (room temperature, pH 7.6), were estimated from the respective gel-shift experiments.

### Surface plasmon resonance

The BIAcore 3000 apparatus (GE Healthcare) used for surface plasmon resonance (SPR) was applied to investigate protein-DNA interactions in real-time. Linear *oriC*-DNA (0.25 pmol, 624 nt) was biotinylated both at its 5´ and 3´ ends (standard PCR with biotinylated primers) and non-covalently bound to a streptavidin coated sensor chip (~1700 RU, Sensor chip SA, GE Healthcare). The system was preequilibrated and permanently flushed (flow rate 20 μl/min) at room temperature with SPR-binding buffer (50 mM HEPES, 300 mM NaCl, 2.5 mM MgCl_2_, pH 7.6) containing or lacking ATP (2.5 mM). (His)_6_-wild type/mutant DnaA (2.5 μM) preincubated for one minute with or without 2.5 mM ATP in SPR-binding buffer was subsequently applied to the sensor chip (volume 75 μl) at a flow rate of 20 μl/min, i.e. for 225 seconds, followed by protein dissociation from the DNA. Protein-DNA interactions were measured in real-time over a period of 700 seconds. The response of the interaction of the protein to the *oriC*-DNA containing chamber was subtracted from unspecific binding to the chip surface monitored in a second DNA-free chamber. SPR-wash buffer (100 mM NaOH, 500 mM NaCl) was injected to remove bound proteins from the chip.

### Fluorescence and FRAP microscopy

Cells were grown in S7_50_ (*B*. *subtilis*) or M9 (*E*. *coli*) minimal medium until they reached mid-exponential phase. To ensure continuous nutrition supply and to immobilize the cells they were covered with a pad consisting of S7_50_ minimal medium and 1% (w/v) agarose. For microscopy, an Olympus BX51 microscope with a digital CCD camera (Cool Snap EZ, Photometrics) controlled by the Metamorph 6.3-Software (Meta Imaging Software) was used.

For FRAP experiments a Zeiss Axio Observer A1 with a TIRF objective (100x, immersion oil, NA: 1.45, Zeiss) was used. Images were acquired using a digital EMCCD camera (Evolve, Photometrics). A 515 nm laser (Visitron Systems, Munich) was used to excite and bleach the sample. Acquisition was controlled using the VisiView 2.1.4 software (Visitron Systems, Munich). A custom made macro was applied for acquiring the FRAP sequences. Analysis of FRAP data was performed using Fiji ImageJ [50] and as described by Kleine Borgmann [31]. Acquired streams were aligned using the StackReg plugin. Fluorescence intensities of the region of interest were measured and background fluorescence subtracted. To correct for acquisition bleaching the fluorescence of a control cell was measured and used to normalize the data (single normalization) [51]. Data were additionally normalized to their pre-bleach levels. In order to facilitate fitting, clusters of three individual cells with similar intensities were grouped together, full-scale normalized to correct for different bleach depths and averaged. Fits to the post-bleach data points of each cluster were obtained using the nonlinear regression algorithm from Wolfram Mathematica 8.0. As a model, the function $f\left(t\right)=A\left(1-e^{-k_{r}t}\right)$, with recovery rate *k*_*r*_ and recovery level *A*, was used. The recovery half time $t_{1/2}=\frac{Ln\;2}{k_{r}}$ was calculated for each cluster. Then the mean of the recovery half times and its standard error were computed. In the plots, one single experiment is visualized together with the corresponding fit. The Student’s t-test was used to compare the means of the recovery halftimes. In order to determine if an immobile fraction was present, we performed double normalization to whole cell intensity [51]. Data from individual cells were normalized by the whole intensity. This corrects for both acquisition bleaching and the initial laser pulse. Proceeding as above, the recovery level *A*, can then be interpreted as the fraction of molecules which are mobile on the timescale of the experiment.

### Single molecule microscopy

For single molecule microscopy an Olympus IX71 with an Olympus TIRF objective (100x, ApoN, NA: 1.7) was used. Image acquisition was accomplished using a back-illuminated EMCCD camera (Andor, iXon Ultra 897). The centre of a 20 fold expanded beam from a 100 mW multiline argon laser (JDS Uniphase, laser head: 2219-G5MLS) was focused on the back-focal plane and operated during image acquisition with 150 to 200 W/cm^2^. An appropriate YFP-laser filter-set was used. For image acquisition the program Andor Solis 4.21 was applied. Streams of 1500 frames of 41 ms or 24.5 Hz were acquired. For faster image acquisition only a subset of 128 × 128 pixel of the chip was read out. High refractive index glass cover slips (n = 1.78) for the Olympus objective were used. Of note, cells continued to grow after imaging, showing that there is little to no photodamage during imaging.

Acquired streams were loaded into Fiji ImageJ [50] and pixel sizes (100 nm) and time increments were calibrated. Tracking of single molecules was achieved using the ImageJ plugin MtrackJ, or u-track 2.0 [52]. Trajectory x/y-coordinates were imported in SMMtrack and parameters like the apparent diffusion constant and the mean square displacement were calculated. SMMtrack (https://github.com/SMMTrack) is a Delphi executable that reads trajectory-data and analyzes the increase of the mean square displacement (MSD). It performs a mean weighted fit on MSD curves of either a whole data set, or just individual trajectories. Also it visualizes individual tracks in superposition with its “heat map”, where each time point in the trajectory emits a constant amount of “heat” into its neighborhood, which will ultimately accumulate to higher degrees of temperatures (= darker shades of red) over time. In order to distinguish real trapping events from trajectories crossing over an additional cooling effect is employed by adding a bell-shaped heat mask for the current trajectory point to a damped version of the previous temperature distribution. The final heat map shows the point wise maximal temperatures of the whole trajectory. SMMtrack also analyzes trapping events within individual trajectories. Trapping events were defined as the time interval in which the trajectory stays within a threshold radius around some trajectory point. This threshold radius is determined by the resolution limit of 230 nm of our microscopic setup. The trapping time distributions in Fig 7 and S10 Fig were derived from disjoint trapping intervals of observed trajectories. Also histogram data was generated of the x/y-proportions of the single step lengths. To estimate the probability that a freely diffusing molecule will be erroneously counted as static we proceed as follows. Firstly, to make the calculation analytically tractable, we allow the bounding circle defining the region in which jumps are considered static to move with the track i.e. we consider individual displacements rather than the net displacement starting from an initial time point as used above. The following false positive rate is therefore likely a slight overestimate. The probability that a molecule with diffusion constant *D* diffuses less than *r*_*max*_ within one frame interval Δt is given by

$$p=1-e^{-\frac{r_{max}^{2}}{4D\Delta t}}.$$

Then the probability that a track of length *n* contains at least 3 consecutive static frames is then given by

P(n)=∑r=3n(n-2n-r)pr(1-p)n-r,

where the binomial coefficient is the number of ways to arrange *n-r* non-static jumps in a track of length *n*-2 (3 consecutive static jumps having being grouped together). Given our distribution of tracks lengths, we can use this result to estimate the false positive rate. For YFP-DnaA, we find a false positive rate of 9%.

## Supporting information

S1 Fig Western blot analyses of DnaA expression levels. Western blot analysis of exponentially growing *B*. *subtilis* cells. Cell extracts were normalized to optical density, anti-DnaA antiserum was used. A) lane 1, wild tye cells, lane 2–4 YFP-DnaA expressed from the xylose promoter at original locus, lane 2: 0.1% xylose, lane 3: 0.2% xylose, lane 4: 0.5% xylose. B) Lanes 1 to 3: wild type cells, lanes 4 to 6: cells expressing YFP-DnaA from the *amyE* locus, under control of the xylose promoter. Lanes 1 and 4: addition of 0.1% xylose for 30 minutes (experimental conditions used in SMT experiments), lanes 2 and 5: 0.5% xylose for 60 minutes, lanes 3 and 6: cells growing in the presence of 0.5% xylose from inoculation. Lower bands DnaA, upper bands YFP-DnaA.
(JPG)

S2 Fig Cell-cycle dependent localization of YFP-YabA. A) Position of YFP-YabA foci to the nearest cell pole in correlation to total cell length. Green circles, single YFP-YabA foci; open triangles, YFP-YabA foci in cells with two foci (the one closest to the cell pole was set nearest to the origin of the x-axis). Dashed line, cell center; Black line, cell length. B)-H) Different localization pattern of YFP-YabA, dependent on cell length. Percentage of cells not showing one of the three signals is not stated (i.e. is the remaining % up to 100%). YFP-YabA (green) localization compared to the origin of replication (red, tagged with LacI-CFP which binds to a *lacO* array in origin region) and the replication machinery (red, DnaX, τ subunit of DNA polymerase III). White line, cell borders; scale bars, 2 μm. I-J) Position of origin regions (I) or DnaX-mCherry foci (J) and YFP-YabA foci to nearest cell pole, dependent on cell length. YFP-YabA symbols are as described in A; Black open diamonds, origin-CFP foci (I) or DnaX-mCherry foci (J).
(JPG)

S3 Fig FRAP analysis of LacI-GFP binding to a lacO array. A) FRAP analysis of cells expressing GFP-LacI binding to a lacO array at 359° on the chromosome. B) FRAP curves of 11 experiments. C) FRAP analysis of cells expressing YFP-DnaA at reduced level (0.2% xylose, P_*xyl*_-*yfp*-*dnaA* at original locus), for comparison see lanes 1 and 2 in S1A Fig.
(JPG)

S4 Fig FRAP measurements of YFP-DnaA in a strain carrying a *lacO* array close to *oriC*. A) Upper panels: cells expressing YFP-DnaA and having *oriC* decorated with LacI-CFP, triangle in overlay indicated YFP-DnaA focus co-localizing with an *oriC* region. Lower panels: FRAP sequence of YFP-DnaA, showing recovery of the fluorescence signal in the region of interest over time. White triangle, region of interest. White dashed circle, area bleached. White lines, cell borders; scale bar 2 μm. B) Upper panels: cells expressing YFP-DnaA and having *oriC* decorated with LacI-CFP, triangle in overlay indicated YFP-DnaA focus not colocalizing with an *oriC* region. Lower panels: FRAP sequence of YFP-DnaA, showing recovery of the fluorescence signal in the region of interest over time. White triangle, region of interest. White dashed circle, area bleached. White lines, cell borders; scale bar 2 μm. C) Fluorescence intensity (%) corrected for general bleaching plotted over time (s). Diagram displays data obtained from a single experiment shown in (A). Red line represents fit used to calculate the recovery half-time. The calculated recovery half-time for YFP-DnaA determined from 10 experiments is 2.7 ± 0.5 s (SEM). D) Evaluation of experiment shown in panel B), half-time recovery for non-origin bound YFP-DnaA is 3.08 ± 0.5 (SEM) from 12 experiments.
(TIF)

S5 Fig Single molecule microscopy of YFP-DnaA. A) Single frame taken from a SMT movie. A single YFP signal is indicated by a circle. The frame is taken after frame 100 shown in panel B), where the corresponding signal is boxed in red. The signal bleaches later in a single step, similar to other signals earlier and later during the experiment. At the beginning of the acquisition, fluorescence bleaches, until single signals are apparent. C) Example of a stream showing several static tracks. D) Heat map of the lower static focus seen in panel (C), E) Graph showing the distance moved from the original start point (black line), and the increments in distance travelled.
(JPG)

S6 Fig Tracking of YFP-DnaA expressed from A) the amylase locus using 0.01% xylose at 25 Hz, and B) as sole source of the protein at 100 Hz, but at lower levels (0.1% xylose) compared to the wild type (see lanes 1 and 2, S1A Fig). A) A single Gaussian fit to the step size distribution reveals an incomplete description of the data (D* = 0.68 μm^2^/s). B) Step size distribution of YFP-DnaA expressed as sole source of the protein fitted by a multivariate Gaussian assuming two populations (D_1_* = 0.2 μm^2^/s (30%) and 1.7 μm^2^/s (70%)). C) Distribution function of squared displacements. The plot shows the probability that a molecule will move a radius *r* in the time *t*. The 3-species fit does not significantly improve the description of the data as seen in the residuals below the plot.
(TIF)

S7 Fig Tracking of free mNeon molecules imaged at 250 Hz in live *E*. *coli* cells. A) Tracks superimposed on sum of individual movie frames. B) Probability density distribution of steps taken by the tracks (n = 889) in the x- and y-plane. A single normal distribution was fitted yielding a diffusion coefficient of 3.3 μm^2^/s. Average lifetime of a mNeon molecule is 27 ms at an illumination power of ~1 kW/cm^2^.
(TIF)

S8 Fig Histograms showing percentage of molecules stopping for 3, 4, 5, 6 and 15 time intervals. One interval corresponds to 0.041 s. A) YFP-DnaA and YFP-YabA, B) YFP-DnaA in wild type and in *soj/spo0J* mutant cells. C) YFP-DnaA and YFP-DnaAE183Q, D) YFP-DnaA and Spo0J-YFP. E) *B*. *subtilis* YFP-DnaA and *E*. *coli* DnaA-YFP^sw^.
(TIF)

S9 Fig Representative heat maps of A) DnaA-YFP^sw^ (in *E*. *coli*) and of B) Spo0J-YFP (in *B*. *subtilis*). (JPG)

S10 Fig Cumulative density function distribution for residence times using tracks longer than 10 time points. A) *amyE*::*yfp-dnaA* (ME15) vs. *amyE*::*yfp-yabA* (KS167). B) *amyE*::*yfp-dnaA* (ME15) vs. *amyE*::*spo0J-yfp*. C) *amyE*::*yfp-dnaA* (ME15) vs. *amyE*::*yfp-dnaA Δsoj-spo0J* (KS192). D) *amyE*::*yfp-dnaA* (ME15) vs. *amyE*::*yfp-dnaAE183Q* (ME20). E) *amyE*::*yfp-dnaA* (ME15) vs. DnaA-YFP^sw^ *E*. *coli* (original locus). Please refer to S1 Table for the average residence times.
(TIF)

S1 Table (PDF)

S1 Movie Movement of mobile YFP-DnaA molecules in *B*. *subtilis* cells tracked at an interval of 41 ms. For clarity the tracks are shown at a rate of 10 frames/s.
(AVI)

S2 Movie Movement of a mobile YFP-DnaA molecule (trajectory shown in blue) in *B*. *subtilis* cells tracked at an interval of 41 ms. For clarity the tracks are shown at a rate of 10 frames/s.
(AVI)

S3 Movie Movement of an immobile YFP-DnaA molecule in a *B*. *subtilis* cell tracked at an interval of 41 ms. For clarity the track is shown at a rate of 10 frames/s.
(AVI)

S4 Movie Movement of a single mNeonGreen molecules in *B*. *subtilis* tracked at an interval of 4 ms. For clarity, the track is shown at 10 frames/s.
(AVI)

S5 Movie Oscillation of DnaA-YFP^sw^ in *E*. *coli* cells. 200 ms stream acquisitions, 5 frames/s.
(AVI)

S6 Movie Realtime movement of single DnaA-YFP^sw^ in an *E*. *coli* cell. 41 ms stream acquisition, shown are 25 frames/s.
(AVI)
