# Supplementary figures and images for: Rapid turnover of DnaA at replication origin regions contributes to initiation control of DNA replication
Source: PLoS Genet. 2017 Feb 6;13(2):e1006561. doi: 10.1371/journal.pgen.1006561 (PMC5319796; doi:10.1371/journal.pgen.1006561)

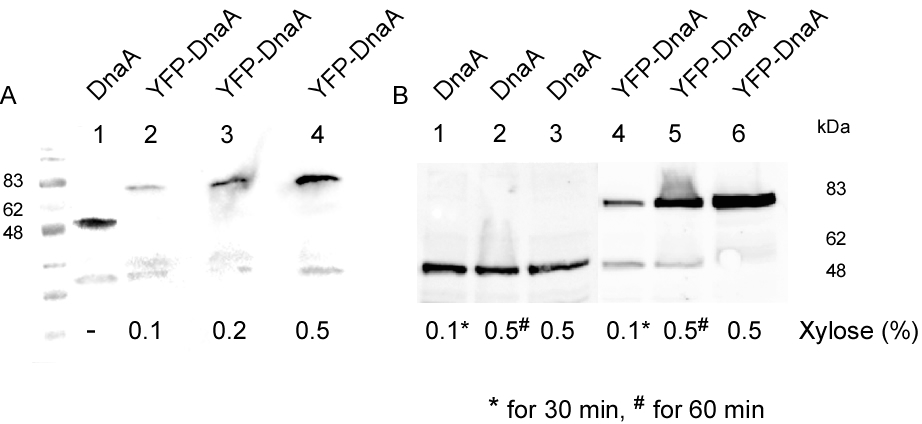

Supplement: S1 Fig — Western blot analysis of exponentially growing B. subtilis cells. Cell extracts were normalized to optical density, anti-DnaA antiserum was used. A) lane 1, wild tye cells, lane 2–4 YFP-DnaA expressed from the xylose promoter at original locus, lane 2: 0.1% xylose, lane 3: 0.2% xylose, lane 4: 0.5% xylose. B) Lanes 1 to 3: wild type cells, lanes 4 to 6: cells expressing YFP-DnaA from the amyE locus, under control of the xylose promoter. Lanes 1 and 4: addition of 0.1% xylose for 30 minutes (experimental conditions used in SMT experiments), lanes 2 and 5: 0.5% xylose for 60 minutes, lanes 3 and 6: cells growing in the presence of 0.5% xylose from inoculation. Lower bands DnaA, upper bands YFP-DnaA. (JPG) [file pgen.1006561.s001.jpg]

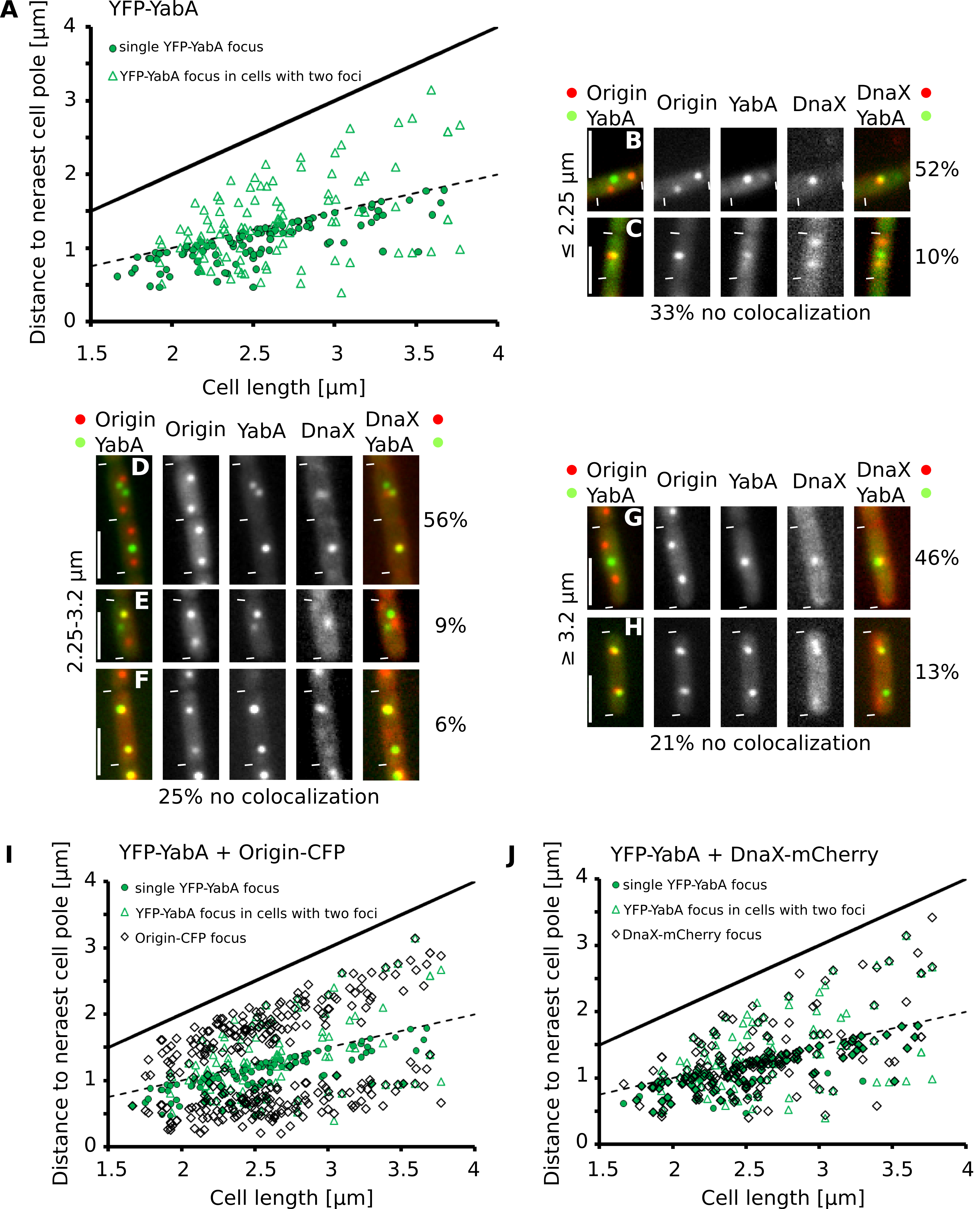

Supplement: S2 Fig — A) Position of YFP-YabA foci to the nearest cell pole in correlation to total cell length. Green circles, single YFP-YabA foci; open triangles, YFP-YabA foci in cells with two foci (the one closest to the cell pole was set nearest to the origin of the x-axis). Dashed line, cell center; Black line, cell length. B)-H) Different localization pattern of YFP-YabA, dependent on cell length. Percentage of cells not showing one of the three signals is not stated (i.e. is the remaining % up to 100%). YFP-YabA (green) localization compared to the origin of replication (red, tagged with LacI-CFP which binds to a lacO array in origin region) and the replication machinery (red, DnaX, τ subunit of DNA polymerase III). White line, cell borders; scale bars, 2 μm. I-J) Position of origin regions (I) or DnaX-mCherry foci (J) and YFP-YabA foci to nearest cell pole, dependent on cell length. YFP-YabA symbols are as described in A; Black open diamonds, origin-CFP foci (I) or DnaX-mCherry foci (J). (JPG) [file pgen.1006561.s002.jpg]

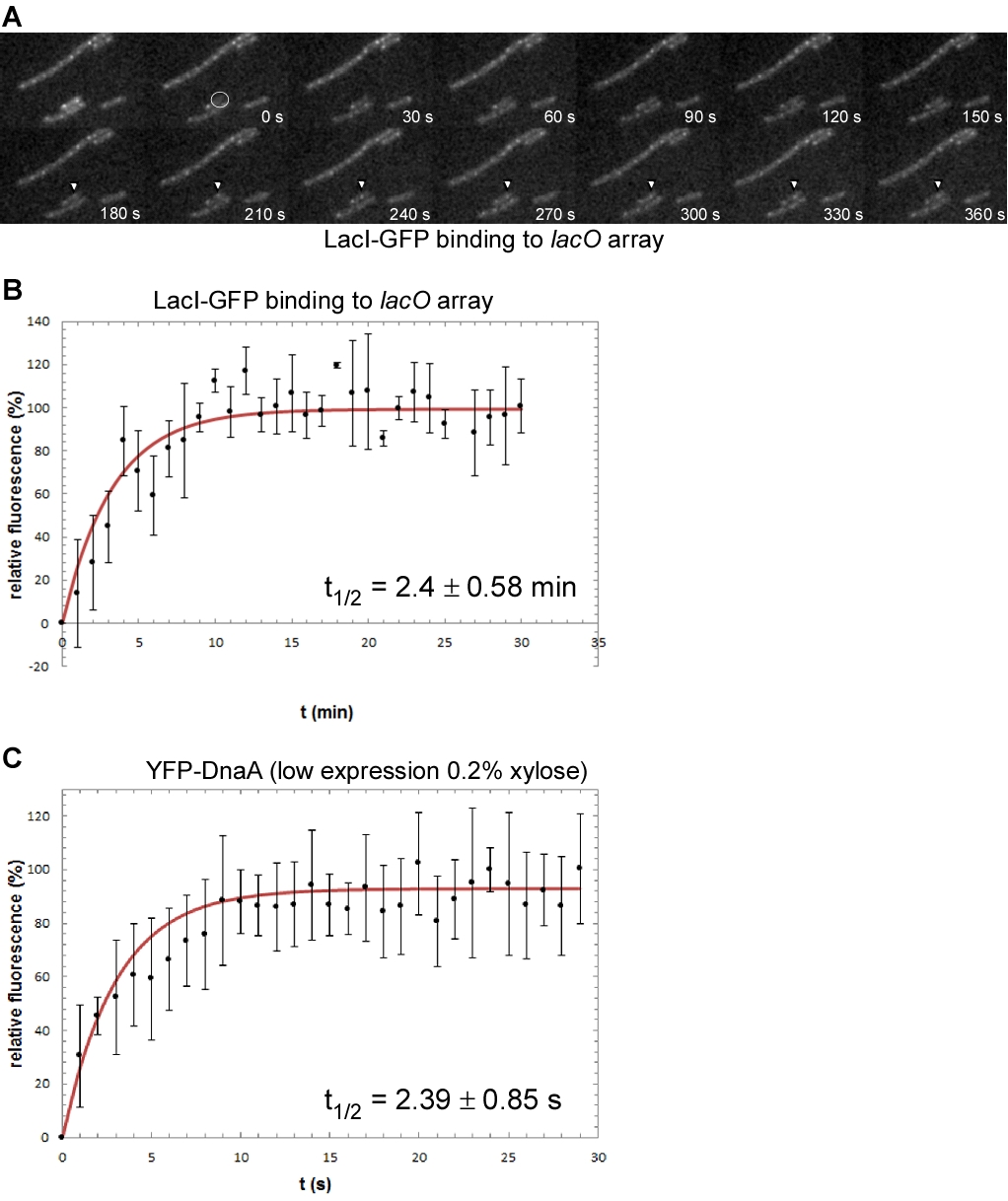

Supplement: S3 Fig — A) FRAP analysis of cells expressing GFP-LacI binding to a lacO array at 359° on the chromosome. B) FRAP curves of 11 experiments. C) FRAP analysis of cells expressing YFP-DnaA at reduced level (0.2% xylose, Pxyl-yfp-dnaA at original locus), for comparison see lanes 1 and 2 in S1A Fig. (JPG) [file pgen.1006561.s003.jpg]

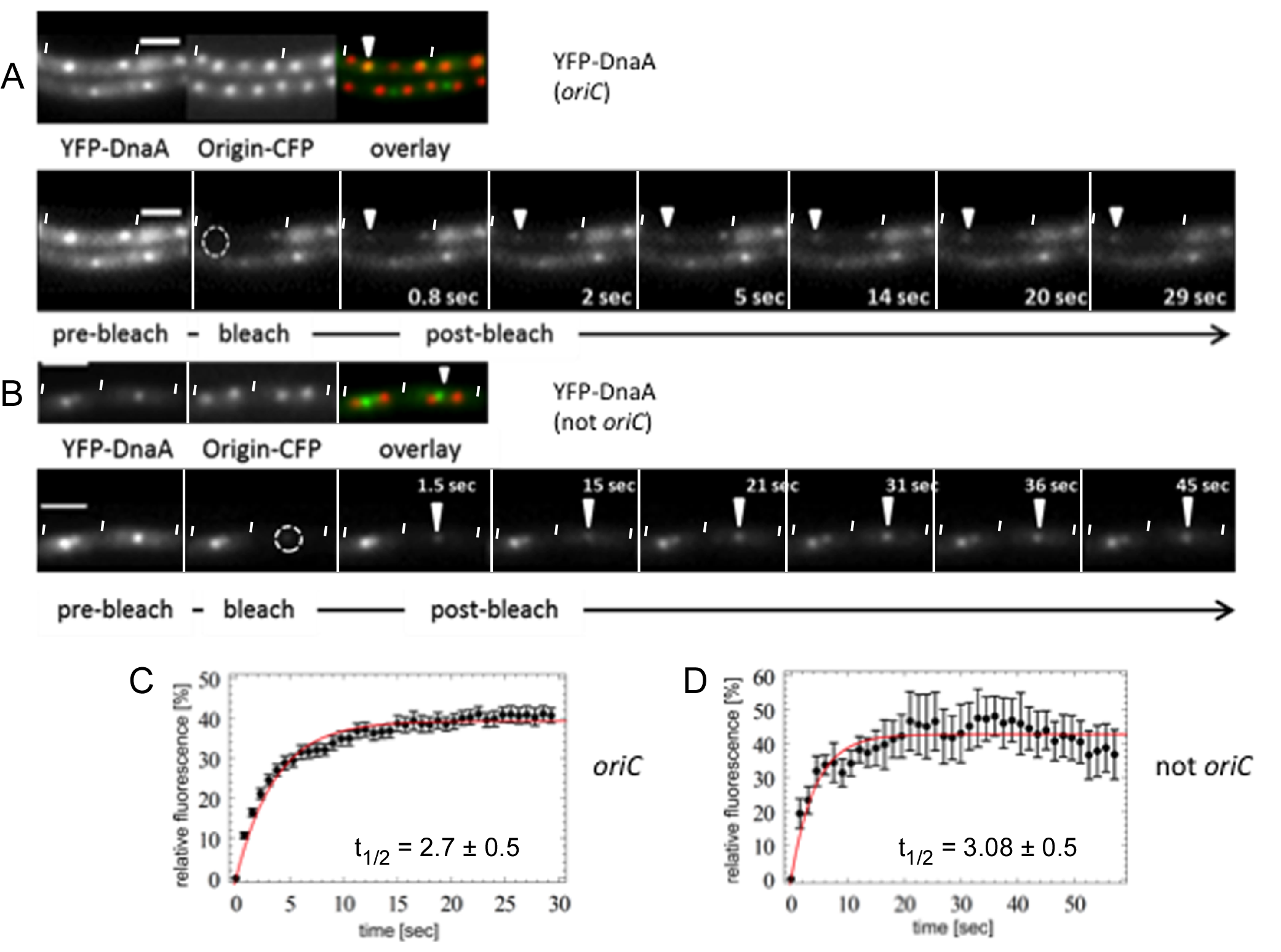

Supplement: S4 Fig — A) Upper panels: cells expressing YFP-DnaA and having oriC decorated with LacI-CFP, triangle in overlay indicated YFP-DnaA focus co-localizing with an oriC region. Lower panels: FRAP sequence of YFP-DnaA, showing recovery of the fluorescence signal in the region of interest over time. White triangle, region of interest. White dashed circle, area bleached. White lines, cell borders; scale bar 2 μm. B) Upper panels: cells expressing YFP-DnaA and having oriC decorated with LacI-CFP, triangle in overlay indicated YFP-DnaA focus not colocalizing with an oriC region. Lower panels: FRAP sequence of YFP-DnaA, showing recovery of the fluorescence signal in the region of interest over time. White triangle, region of interest. White dashed circle, area bleached. White lines, cell borders; scale bar 2 μm. C) Fluorescence intensity (%) corrected for general bleaching plotted over time (s). Diagram displays data obtained from a single experiment shown in (A). Red line represents fit used to calculate the recovery half-time. The calculated recovery half-time for YFP-DnaA determined from 10 experiments is 2.7 ± 0.5 s (SEM). D) Evaluation of experiment shown in panel B), half-time recovery for non-origin bound YFP-DnaA is 3.08 ± 0.5 (SEM) from 12 experiments. (TIF) [file pgen.1006561.s004.tif]

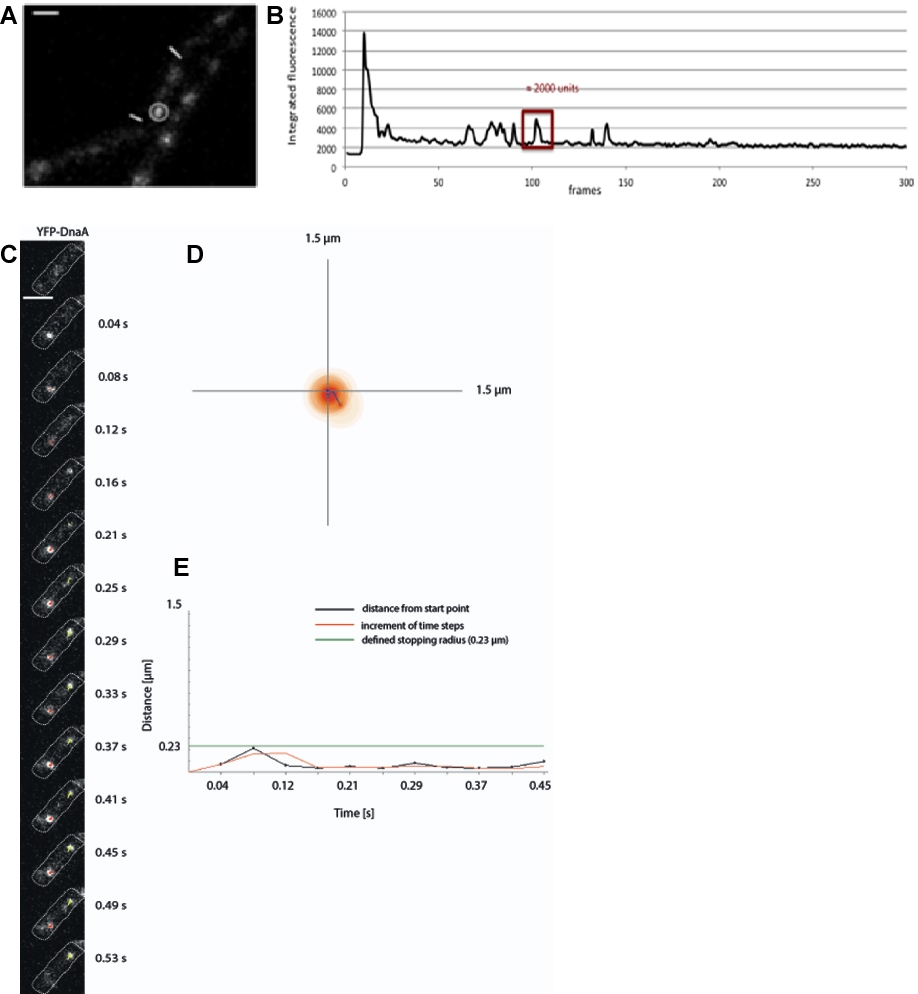

Supplement: S5 Fig — A) Single frame taken from a SMT movie. A single YFP signal is indicated by a circle. The frame is taken after frame 100 shown in panel B), where the corresponding signal is boxed in red. The signal bleaches later in a single step, similar to other signals earlier and later during the experiment. At the beginning of the acquisition, fluorescence bleaches, until single signals are apparent. C) Example of a stream showing several static tracks. D) Heat map of the lower static focus seen in panel (C), E) Graph showing the distance moved from the original start point (black line), and the increments in distance travelled. (JPG) [file pgen.1006561.s005.jpg]

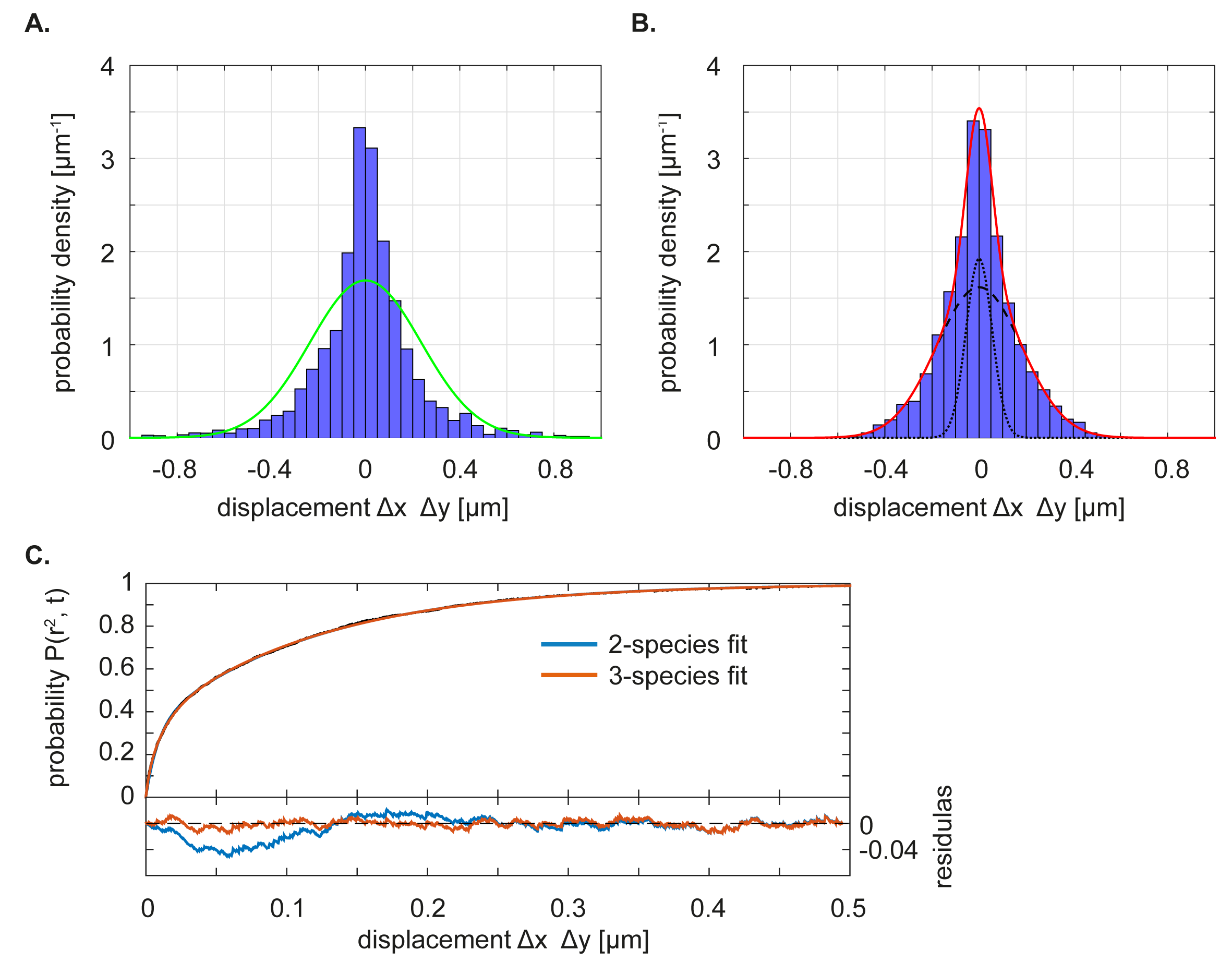

Supplement: S6 Fig — Tracking of YFP-DnaA expressed from A) the amylase locus using 0.01% xylose at 25 Hz, and B) as sole source of the protein at 100 Hz, but at lower levels (0.1% xylose) compared to the wild type (see lanes 1 and 2, S1A Fig). A) A single Gaussian fit to the step size distribution reveals an incomplete description of the data (D* = 0.68 μm2/s). B) Step size distribution of YFP-DnaA expressed as sole source of the protein fitted by a multivariate Gaussian assuming two populations (D1* = 0.2 μm2/s (30%) and 1.7 μm2/s (70%)). C) Distribution function of squared displacements. The plot shows the probability that a molecule will move a radius r in the time t. The 3-species fit does not significantly improve the description of the data as seen in the residuals below the plot. (TIF) [file pgen.1006561.s006.tif]

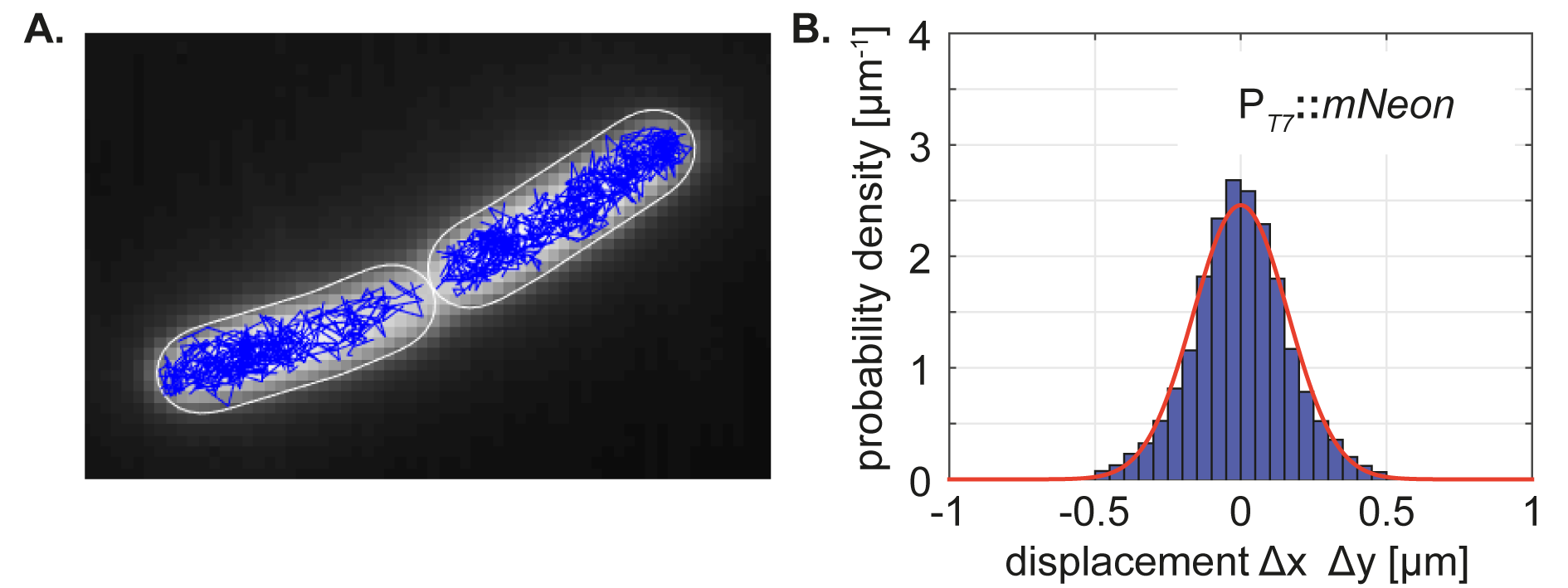

Supplement: S7 Fig — A) Tracks superimposed on sum of individual movie frames. B) Probability density distribution of steps taken by the tracks (n = 889) in the x- and y-plane. A single normal distribution was fitted yielding a diffusion coefficient of 3.3 μm2/s. Average lifetime of a mNeon molecule is 27 ms at an illumination power of ~1 kW/cm2. (TIF) [file pgen.1006561.s007.tif]

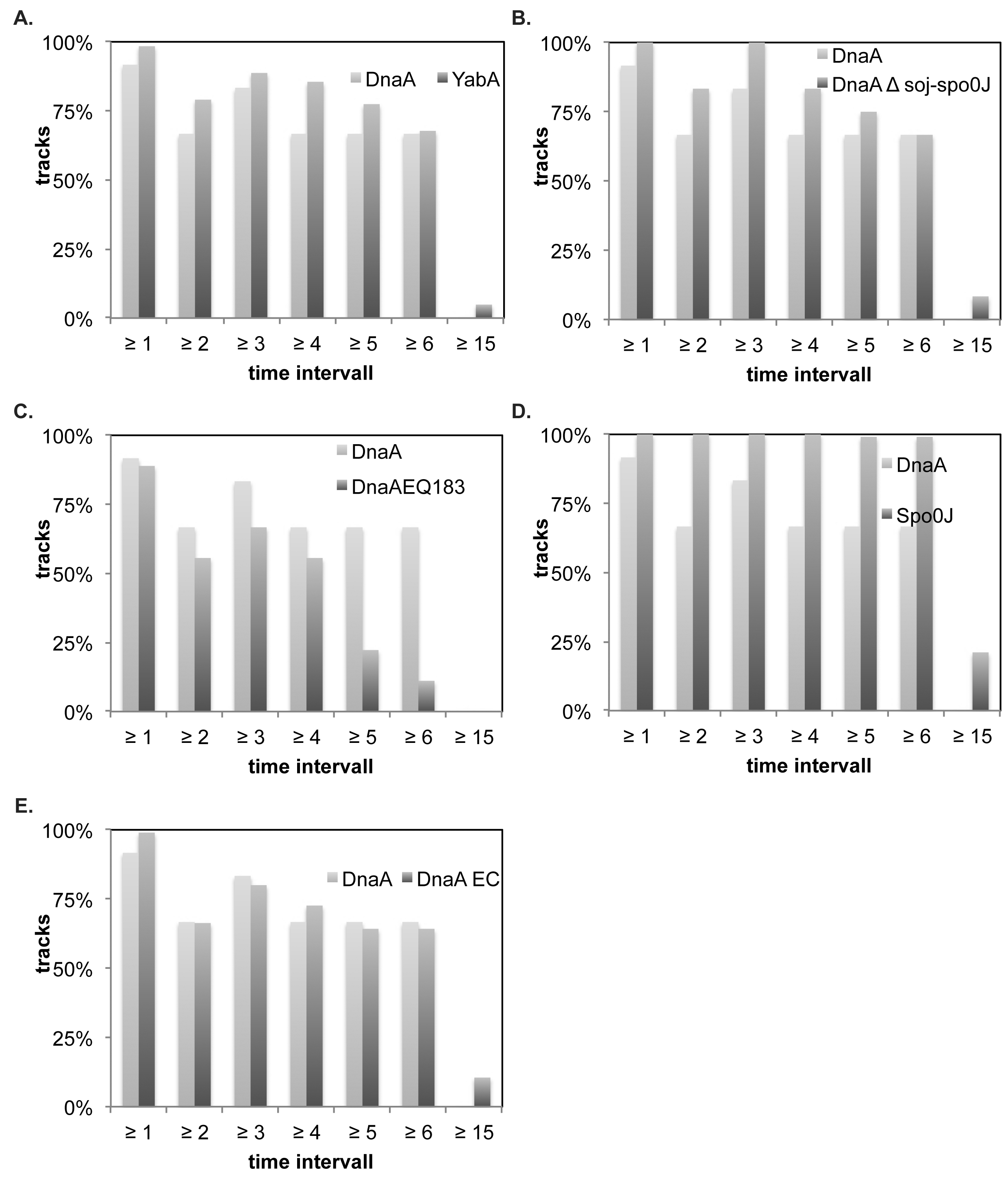

Supplement: S8 Fig — One interval corresponds to 0.041 s. A) YFP-DnaA and YFP-YabA, B) YFP-DnaA in wild type and in soj/spo0J mutant cells. C) YFP-DnaA and YFP-DnaAE183Q, D) YFP-DnaA and Spo0J-YFP. E) B. subtilis YFP-DnaA and E. coli DnaA-YFPsw. (TIF) [file pgen.1006561.s008.tif]

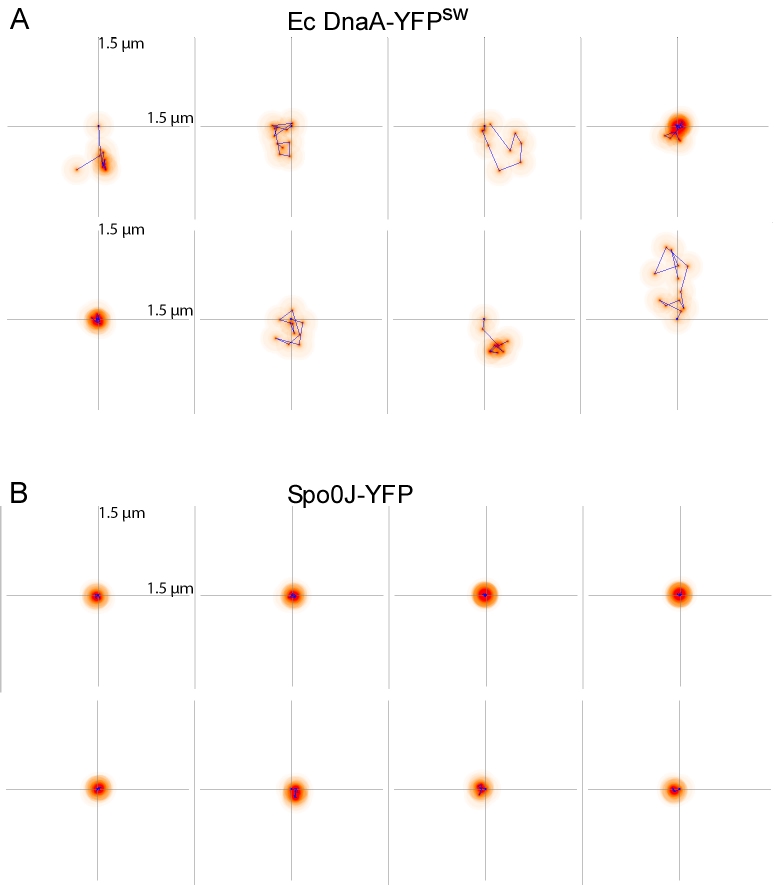

Supplement: S9 Fig — (JPG) [file pgen.1006561.s009.jpg]

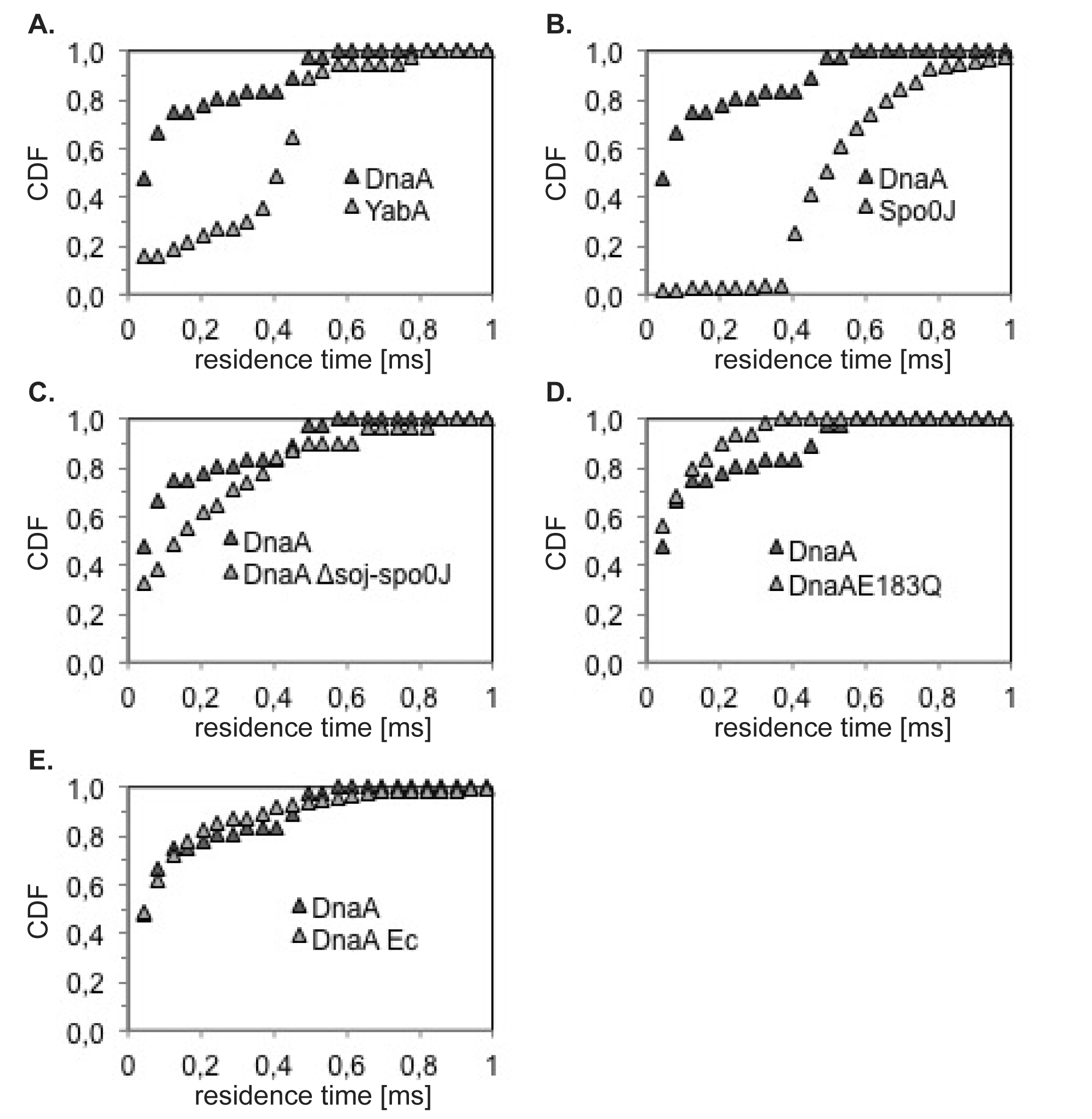

Supplement: S10 Fig — A) amyE::yfp-dnaA (ME15) vs. amyE::yfp-yabA (KS167). B) amyE::yfp-dnaA (ME15) vs. amyE::spo0J-yfp. C) amyE::yfp-dnaA (ME15) vs. amyE::yfp-dnaA Δsoj-spo0J (KS192). D) amyE::yfp-dnaA (ME15) vs. amyE::yfp-dnaAE183Q (ME20). E) amyE::yfp-dnaA (ME15) vs. DnaA-YFPsw E. coli (original locus). Please refer to S1 Table for the average residence times. (TIF) [file pgen.1006561.s010.tif]
