# Supplementary material for: Rapid turnover of DnaA at replication origin regions contributes to initiation control of DNA replication
Source: PLoS Genet. 2017 Feb 6;13(2):e1006561. doi: 10.1371/journal.pgen.1006561 (PMC5319796; doi:10.1371/journal.pgen.1006561)
Supplement: S1 Table — (PDF) [file pgen.1006561.s011.pdf]

Table S1: residence times for tracks longer than 10 frames

| strain | dwell time [ms] | ♯tracks > 10 frames |
|---|---|---|
| ME15 (*amyE::yfp-dnaA*) | 342 ± 8.3 (SD) | 11 |
| ME20 (*amyE::yfp dnaAE183Q*) | 205 ± 2.4 (SD) | 9 |
| KS167 (*amyE::yfp-yabA* D*yabA*) | 442 ± 9.2 (SD) | 28 |
| KS192 (*amyE::yfp-dnaA*, D*soj-spo0J*) | 345 ± 8.4 (SD) | 28 |
| DnaA-YFP$^{sw}$ *E. coli* (original locus) | 310 ± 3.6 (SD) | 93 |
| Spo0J-YFP | 559 ± 13.4 (SD) | 109 |
